# Neuronal–glial communication perturbations in murine *SOD1*^*G93A*^ spinal cord

**DOI:** 10.1038/s42003-022-03128-y

**Published:** 2022-02-28

**Authors:** Michael MacLean, Raquel López-Díez, Carolina Vasquez, Paul F. Gugger, Ann Marie Schmidt

**Affiliations:** Diabetes Research Program, Department of Medicine, New York University Grossman School of Medicine, New York, NY 10016 USA

**Keywords:** Cellular neuroscience, Amyotrophic lateral sclerosis

## Abstract

Amyotrophic lateral sclerosis (ALS) is an incurable disease characterized by proteinaceous aggregate accumulation and neuroinflammation culminating in rapidly progressive lower and upper motor neuron death. To interrogate cell-intrinsic and inter-cell type perturbations in ALS, single-nucleus RNA sequencing was performed on the lumbar spinal cord in the murine ALS model *SOD1*^*G93A*^ transgenic and littermate control mice at peri-symptomatic onset stage of disease, age 90 days. This work uncovered perturbed tripartite synapse functions, complement activation and metabolic stress in the affected spinal cord; processes evidenced by cell death and proteolytic stress-associated gene sets. Concomitantly, these pro-damage events in the spinal cord co-existed with dysregulated reparative mechanisms. This work provides a resource of cell-specific niches in the ALS spinal cord and asserts that interwoven dysfunctional neuronal-glial communications mediating neurodegeneration are underway prior to overt disease manifestation and are recapitulated, in part, in the human post-mortem ALS spinal cord.

## Abstract (web summary)

In this paper, single-nucleus RNA sequencing was performed to provide a resource of cell-specific niches in the murine ALS model spinal cord at peri-symptomatic onset stage of disease. The data suggest that dysfunctional neuronal-glial communication occurs prior to disease onset, which is partially recapitulated in human post-mortem ALS spinal cord tissue.

## Introduction

Amyotrophic lateral sclerosis (ALS), a disorder of rapidly progressive lower and upper motor neuron death and paralysis, is characterized by disrupted proteostasis, inflammation, and dysregulation of cellular homeostatic functions in the spinal cord; processes that are magnified by maladaptive neuronal–glial communications^1–4^. *SOD1*^*G93A*^ transgenic mice^5,6^ are an established murine model of ALS with predicted gain-of-toxic functions mutations in *SOD1*, such as G93A, which have been identified in patients with familial forms of this disease^7^. Although sporadic forms of ALS are more common, an underlying genetic basis for all cases has been posited, a setting in which environmental and stochastic factors may ultimately trigger presentation of overt disease^8^. There is mounting evidence that ALS is driven by non-cell autonomous alterations^9,10^. In fact, removing the *SOD1* transgene solely from neurons was insufficient to protect from pathology^11–15^. Several studies have reported that modulation of glial or neuronal properties extends lifespan of *SOD1*^*G93A*^ mice^1,4,16–20^. Altogether, these seminal studies provided early evidence that ALS-like phenotypes due to mutant *SOD1* springs from the contributions of multiple cell types. Yet, the precise underlying mechanisms driving these dysfunctional intercellular communications between and within cell types remain unclear.

Accordingly, in the present studies, we considered the use of single-nucleus RNA sequencing (snRNA-seq) versus single-cell RNA-seq (scRNA-seq) to probe the mechanisms of cell-intrinsic and intercellular communications in lumbar spinal cord in a murine model of ALS. In many organs, snRNA-seq achieves comparable gene detection to that of scRNA-seq but has distinct advantages, such as reduced dissociation bias and the elimination of dissociation-induced transcriptional stress responses, as well as compatibility with frozen samples^21^. In the brain tissue in mice, it was reported that although more transcripts were detected in individual whole cells vs. nuclei, both methods were able to discriminate closely-related neuronal cell types. It was concluded that nuclear RNA provided a high degree of information for the characterization of cellular diversity in brain tissues^22^. Furthermore, recent methodological advances have made it possible to label individual nuclei with oligonucleotides, thereby allowing sample pooling, thus further reducing potential batch-associated effects^23^. Thus, we selected snRNA-seq for our studies^23,24^.

Here snRNA-seq was performed on isolated nuclei from lumbar spinal cord tissue of male and female *SOD1*^*G9A3*^ transgenic and littermate control mice at 90 days of age, a peri-symptomatic stage of pathology. Differential gene expression and gene set analyses revealed transcriptional alterations indicative of perturbed glutamatergic synapses, secreted synaptic modulators, complement activation, coincident with dysfunctional repair mechanisms. Specifically, astrocytes displayed transcriptional alterations suggestive of metabolic reprogramming including predicted up-regulation of lipid metabolism-associated transcription factors. In microglia, pathways noted as “systemic lupus erythematosus” and “cytokine-cytokine receptor interaction” were upregulated, suggesting deviation from canonical homeostatic functions. Meanwhile, a population of oligodendrocytes displayed altered transcriptional profiles reminiscent of dysfunctional repair mechanisms. Our findings affirm disrupted cell-intrinsic and neuronal-glial intercellular communications and identify that opposing forces are in play that both exacerbate cellular injury and endeavor to promote repair at a peri-symptomatic stage of pathology. Collectively, this work provides an atlas of cell type-specific alterations emphasizing that these coordinated changes drive neurodegenerative processes and impact intercellular communication; processes that are recapitulated, in part, in the human post-mortem ALS spinal cord.

## Results

### snRNA-seq of *SOD1*^*G93A*^ reveals cell-type-specific transcriptional alterations

**Fig. 1:**
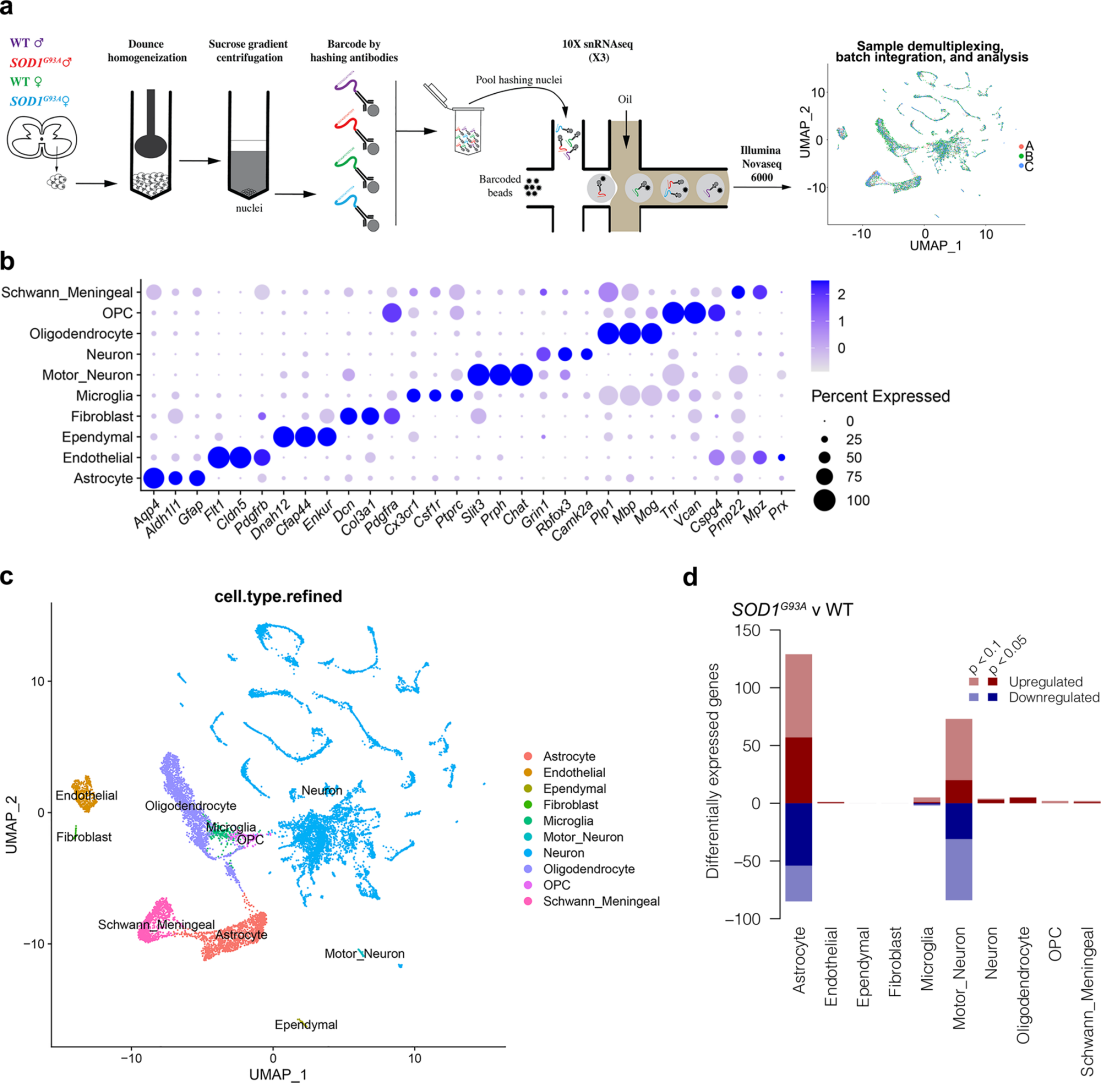
Single-nucleus RNA-seq of murine *SOD1*^*G93A*^ and WT mice lumbar spinal cord captures major cell types at peri-symptomatic onset stage of disease. **a** Experimental design schematic. a-b-c refers to the individual batches in rightmost panel. **b** Dot plot of marker gene expression in each cell type. **c** Dimensionality reduction (UMAP) plot of lumbar spinal cord nuclei colored by assigned cell type. **d** Bar chart summarizing the number of differentially expressed genes (DEGs) between genotypes for each cell type (FDR < 0.1). Positive and negative *y*-axis values indicate number of up-regulated and down-regulated genes, respectively. *N* = 3 independent mice per genotype per sex.

To interrogate the cellular contributions and the impact of intercellular communications, we applied snRNA-seq with nuclear hash-tagging of lumbar spinal cord from male and female *SOD1*^*G93A*^ and littermate control mice at a peri-symptomatic stage of pathology, age 90 days (Fig. 1a) (Supplementary Fig. 1a–f). Transcriptionally defined clusters of nuclei from both genotypes and sexes were identified for neurons, motor neurons, oligodendrocytes, oligodendrocyte precursors (OPCs), astrocytes, microglia, fibroblasts, endothelial cells, ependymal cells, and a heterogeneous population of Schwann cells and other meningeal cells (Fig. 1b, c, Supplementary Fig. 2a, b)^24^. There were no significant differences in the abundance of any one cell type by genotype or sex (FDR > 0.05).

Differential expression analyses between genotypes for each cell type indicated that astrocytes and motor neurons displayed the largest number of differentially expressed genes (DEGs), followed by oligodendrocytes and microglia (Fig. 1d). Other neuronal sub-types displayed few DEGs (Supplementary Fig. 2c, Supplementary Data 1). As expected, *SOD1* was consistently overexpressed in *SOD1*^*G93A*^ cells^5,6^ (Supplementary Data 1). Besides X- or Y-linked genes, such as *Xist*, most cell types did not display gene expression differences between sexes (averaged across genotype) with the exception of microglia (Supplementary Data 1). Four genes demonstrated a significant genotype-by-sex interaction: one gene in astrocytes: *Hpse2*; and three within motor neurons: *Frmpd4, Klhl1*, and *Spint2* (Supplementary Data 1). In the case of these genes, the magnitude and/or direction of expression changes between genotypes differed depending on the sex of the mice.

### In silico modeling of *SOD1*^*G93A*^ intercellular networking reveals dysfunctional communication

**Fig. 2:**
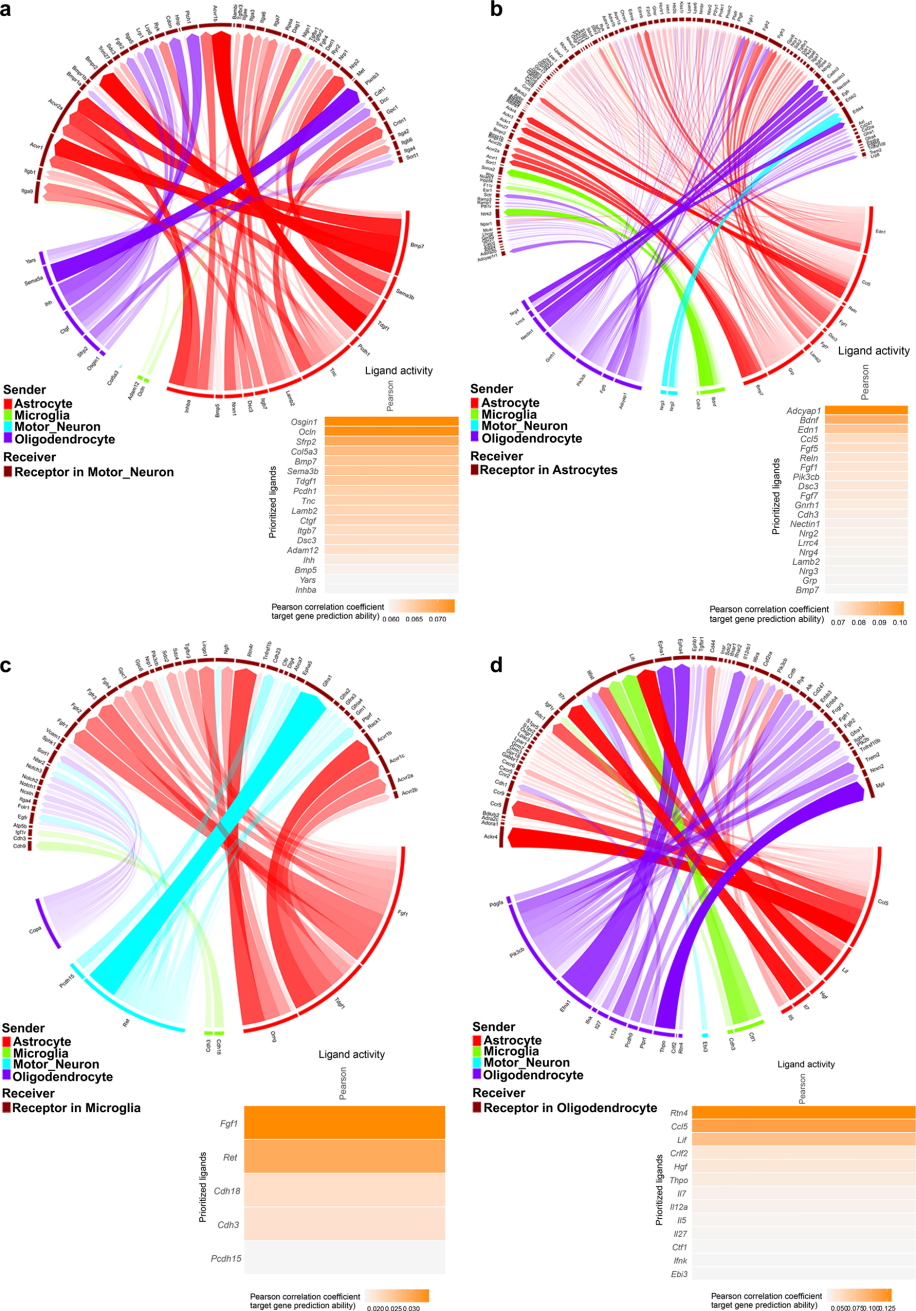
Intercellular communication between motor neurons, astrocytes, microglia and oligodendrocytes is altered between *SOD1*^*G93A*^ and WT cells at peri-symptomatic onset stage of disease. Circos plots^88^ of the top putative ligand–receptor interactions and heatmaps of top ligands’ Pearson correlation coefficients, an estimate of ligand activity, in: **a** motor neurons, **b** astrocytes, **c** microglia, and **d** oligodendrocytes. Line width and opacity indicate ligand–receptor interaction weight. Color legend indicates predicted cellular source of ligand based on maximal expression in included cell types. *N* = 3 independent mice per genotype per sex.

While many studies have investigated discrete roles for individual cell types in the progression of ALS, few, if any studies, have examined the interdependencies of these cellular interactions and their alterations in disease. As the most transcriptional alterations during this peri-symptomatic onset period were identified in motor neurons, microglia, astrocytes, and oligodendrocytes, we focused on these cell types for further analyses. NicheNetR was employed to examine potential cellular cross-talk by inferring receptor-ligand pairs, which could drive the observed differential expression patterns among these cells. Many of the top predicted ligands across cell types related to extracellular matrix and cell–cell adhesion molecules, including those present at synapses such as neuroregulins, Nogo, and cadherins (Fig. 2a–d). Altogether, this analysis suggests dysregulation of motor neuron–glia interactions, potentially at synapses. Accordingly, we examined the specific transcriptomic and transcription factor regulatory nodes within each of these cell types, beginning with motor neurons, to establish evidence for this putative model.

### Motor neurons undergo cell death- and synaptic dysfunction-related transcriptional changes in *SOD1*^*G93A*^ mice at age 90 days

**Fig. 3:**
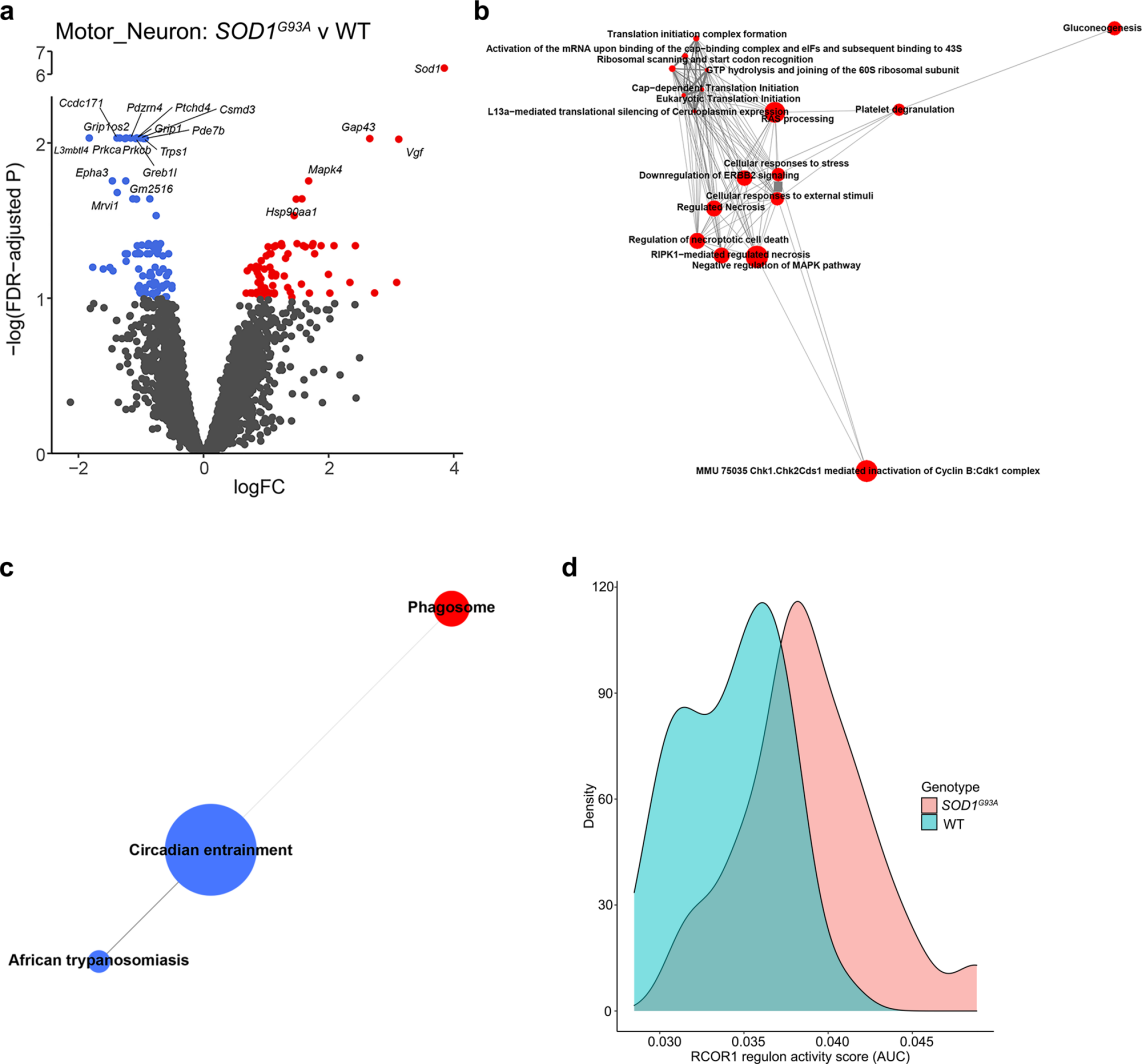
*SOD1*^*G93A*^ motor neurons exhibit altered synaptic functions concomitant with increased cell death-associated pathways at peri-symptomatic onset stage of disease. **a** Volcano plot of differential expression between genotypes in motor neurons. Genes with FDR < 0.1 are colored red (up-regulated) or blue (down-regulated). **b** Motor neuron Reactome gene sets that are significantly differentially expressed between genotypes in both ROAST and CAMERA displayed as a network. **c** Motor neuron KEGG gene sets that are significantly differentially expressed between genotypes in both ROAST and CAMERA displayed as a network. For **b**, **c**: Internode line thickness reflects number of shared genes. Node size represents number of genes in the set. Red and blue nodes denote up-regulation and down-regulation, respectively. **d** Density plot of RCOR1 regulon activity scores for motor neurons. *N* = 3 independent mice per genotype per sex.

Neuronal stress responses in ALS are exacerbated by the intracellular collection of proteinaceous aggregates and through damaging signals emitted by other cell types^4,25,26^. In motor neurons, 158 DEGs, with false-discovery rate (FDR) < 0.1, were identified between *SOD1*^*G93A*^ and wild-type (WT) mice (Fig. 3a, Supplementary Data 1). To examine the overarching KEGG and Reactome gene sets that exhibited the strongest alterations, we utilized self-contained rotation (ROAST) and competitive correlation-adjusted mean rank (CAMERA) gene set testing^27,28^. Cell death and reactive oxygen species (ROS) production-associated gene sets were strongly up-regulated in *SOD1*^*G93A*^ motor neurons (Fig. 3b, c, Supplementary Data 1).

To discover potential regulatory transcription factors underlying the observed differential gene expression, single-cell regulatory network inference and clustering analysis (SCENIC)^29^ was performed. SCENIC generates a list of inferred transcription factors and targets, termed a “regulon”, and calculates an activity score based on ranked expression of the regulon relative to all genes in each cell. This analysis revealed several transcriptional regulators with altered activity in *SOD1*^*G93A*^ motor neurons including dysregulation of the RCOR1 regulon, consisting of downstream genes associated with proteostasis and metabolic pathways (Fig. 3d, Supplementary Fig. 3a, Supplementary Data 1). RCOR1 is a transcriptional and epigenetic regulator of neuronal fate, thereby implying that even before symptomatic onset, motor neuron homeostatic properties are substantively perturbed^30,31^.

Multiple synapse-associated gene sets were significantly altered according to ROAST including “Glutamatergic synapse” (Supplementary Data 1, Supplementary Fig. 3b). In fact, among the DEGs were glutamate receptors *Grip1, Grid2*, and *Grik4*, all of which were significantly down-regulated in *SOD1*^*G93A*^ vs. WT motor neurons. Altogether, these data suggest that motor neurons may exhibit altered glutamatergic synapse function coupled with cell death-associated pathways and pointed to the possibility of altered astrocyte properties.

### *SOD1*^*G93A*^ astrocytes exhibit unique transcriptional changes indicative of altered metabolic, supportive and stress responses at age 90 days

**Fig. 4:**
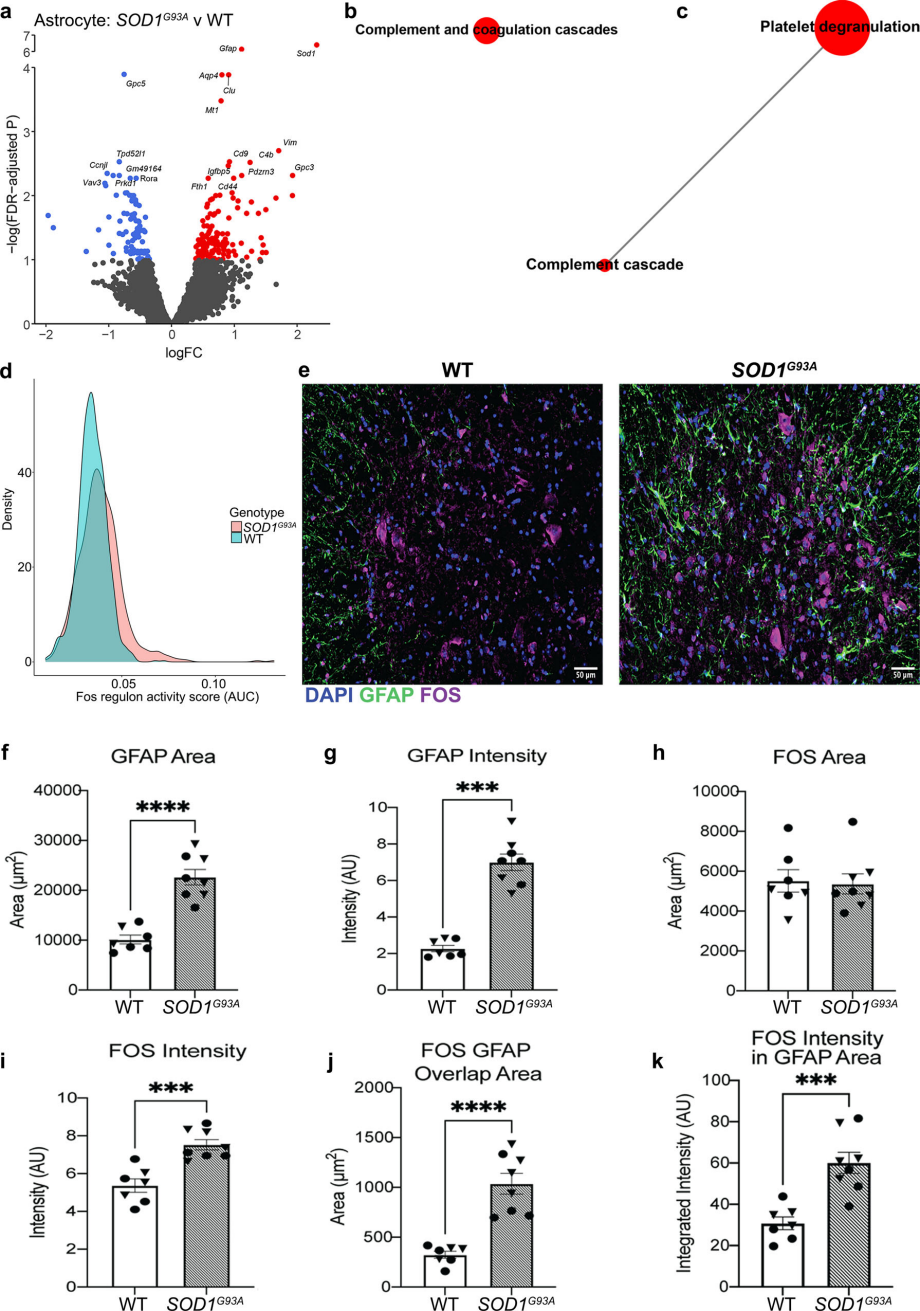
*SOD1*^*G93A*^ astrocytes exhibit altered synaptic support and oxidative stress functions at peri-symptomatic onset stage of disease. **a** Volcano plot of differential expression between genotypes in astrocytes. Genes with FDR < 0.1 are colored red (up-regulated) or blue (down-regulated). Astrocyte (**b**) Reactome and (**c**) KEGG gene sets that are significantly differentially expressed between genotypes in both ROAST and CAMERA displayed as a network. For **b**, **c**: Internode line thickness reflects number of shared genes. Node size represents number of genes in the set. Red nodes denote up-regulation. **d** Density plot of FOS regulon activity scores for astrocytes. **e** Representative images of DAPI, GFAP and FOS in male murine WT and *SOD1*^*G93A*^ lumbar spinal cord at 120 days of age. Quantification of (**f**) GFAP area; (**g**) GFAP intensity; (**h**) FOS area; (**i**) FOS intensity; (**j**) FOS and GFAP overlap area; and (**k**) FOS intensity within GFAP area. In **e**–**k**, *SOD1*^*G93A*^ *N* = 4 independent mice per sex, WT *N* = 3 male and *N* = 4 female mice. Male mice are displayed as triangles, female mice are circles. In **a**–**d**: *N* = 3 independent mice per genotype per sex. In **f**, **h**–**k**, Independent two-sample two-sided *t*-test. In **g**: Mann–Whitney *U*-test. ***p < 0.001, ****p < 0.0001. Specific *p* values are as follows: In **f**, ****p < 0.0001; in **g**, ***p = 0.0003; in **i**, ***p = 0.0003; in **j**, ****p ≤ 0.0001; and in **k**, ***p = 0.0004. Data are presented as mean ± SEM.

Extensive research implicates non-neuronal cells in the pathogenesis of ALS^1–4,32,33^ and pinpoints pathological roles for astrocyte dysfunction in the promotion of neuronal injury and disease progression, at least in part through the loss of support factors, but the full range of mechanisms remains elusive^2,16,34^. Accordingly, *SOD1*^*G93A*^ vs. WT astrocytes exhibited differential expression of 214 genes (Fig. 4a). Up-regulation of *Grin2c*, *Sparc*, and *Clu* was identified; these genes regulate glutamate-induced signal transduction and synapse function (Fig. 4a, Supplementary Data 1)^35–38^. The strongest gene-set alterations within astrocytes were related to complement activation (Fig. 4b, c, Supplementary Data 1). These data align with previous studies that illustrated gene expression differences with inflammatory and metabolic signatures in neurodegeneration and complement induction by reactive astrocytes^16,33,39,40^.

SCENIC analysis revealed that transcriptional activity of inflammation-associated and metabolic transcription factors was altered in *SOD1*^*G93A*^ vs. WT astrocytes (Supplementary Data 1). Among these, FOS was up-regulated and predicted to regulate 16 DEGs downstream, including *Gfap* and *Clu*, (Fig. 4d, Supplementary Fig. 4a), which are linked to metabolism, inflammation and neurodegeneration. Immunofluorescence analysis of *SOD1*^*G93A*^ lumbar spinal cord revealed significantly increased glial fibrillary acid protein (GFAP) area and intensity vs. control spinal cord, which is consistent with *Gfap* as a DEG (Fig. 4e–g). Although the area of FOS staining was not different between genotypes, the intensity was significantly higher in *SOD1*^*G93A*^ spinal cord (Fig. 4e, h, i). Astrocyte-specific FOS expression was higher by both overlap area of GFAP+ and FOS+ and by the integrated intensity of FOS within GFAP area, thereby buttressing the results of this predicted model regarding mechanisms of astrocyte dysfunction (Fig. 4e, j, k).

Furthermore, altered expression of metal homeostasis genes, such as *Fth1*, *Mt1*, *Mt2* and *Mt3*, alongside dysregulated lipid metabolism, suggested altered oxidative stress in astrocytes (Fig. 4a, Supplementary Data 1). In line with this prediction, immunohistochemical analysis revealed increased 4-hydroxynonenal (4-HNE), a lipid peroxidation-induced adduct associated with increased oxidative stress, in the lumbar spinal cord of *SOD1*^*G93A*^ vs. WT mice (Supplementary Fig. 4b–d). GFAP co-localization with 4-HNE revealed increased overlap area and increased integrated intensity of 4-HNE within GFAP area (Supplementary Fig. 4b, e, f). 4-HNE immunostaining was identified in other cell types as well, which supports the transcriptional findings observed in the motor neurons and suggests that they are also under increased oxidative stress (Supplementary Fig. 4b, Fig. 3a). Altogether, these data indicate that inherent metabolic dysfunction within astrocytes may affect their association with synapses, concomitant with altered metabolic and oxidative stress functions.

### Microglia from *SOD1*^*G93A*^ mice exhibit transcriptional changes indicative of complement activation and viral responses at age 90 days

**Fig. 5:**
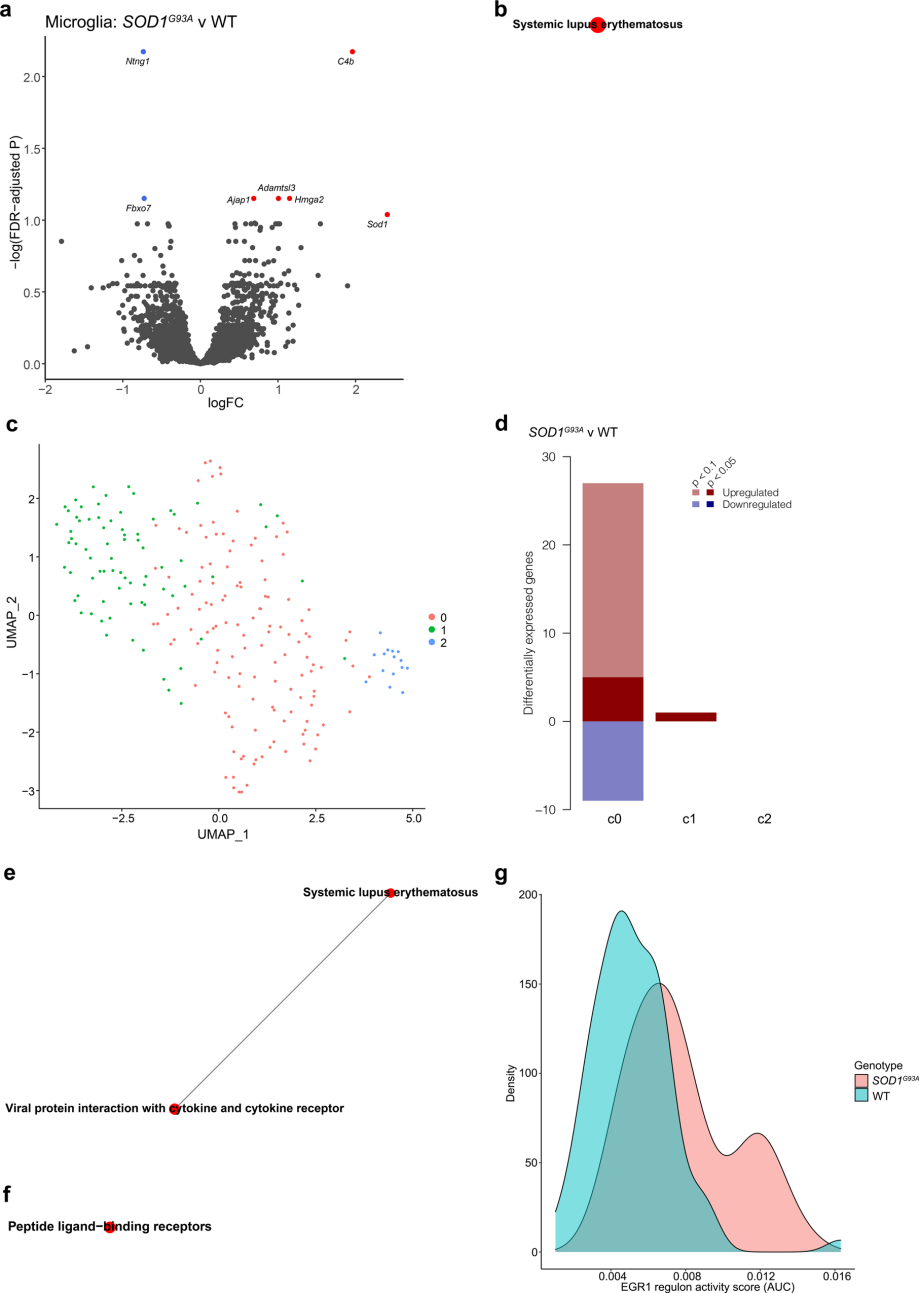
A sub-population of microglia from *SOD1*^*G93A*^ mice exhibits increased immune response pathways at peri-symptomatic onset stage of disease. **a** Volcano plot of DEGs between genotypes in microglia. Genes with FDR < 0.1 are colored red (up-regulated) or blue (down-regulated). **b** Microglia KEGG gene sets that are significantly differentially expressed between genotypes in both ROAST and CAMERA displayed as a network. **c** UMAP plot of microglia sub-clusters. **d** Bar chart summarizing the number of DEGs in each sub-cluster (FDR < 0.1). Positive and negative *y*-axis values indicate number of up-regulated and down-regulated genes, respectively. Sub-cluster 0 microglia KEGG (**e**) and Reactome (**f**) gene sets that are significantly differentially expressed between genotypes in both ROAST and CAMERA displayed as a network. For **b**, **e**, and **f**: Internode line thickness reflects number of shared genes. Node size represents number of genes in the set. Red nodes denote up-regulation. **g** Density plot of EGR1 regulon activity scores for microglia sub-cluster 0. *N* = 3 independent mice per genotype per sex.

Only 7 DEGs were observed between *SOD1*^*G93A*^ and WT microglia, including a member of the complement family, *C4b* (Fig. 5a, Supplementary Data 1). The KEGG pathway “Systemic lupus erythematosus” was significantly altered according to both CAMERA and ROAST (Fig. 5b, Supplementary Data 1). Unlike the other cell types, microglia demonstrated differential expression of 35 genes between the sexes, but expression of these genes did not differ by genotype (Supplementary Data 1). These 35 genes have diverse functions, including calcium homeostasis, neurotransmitter uptake, cell adhesion and metabolic processes; hence, the precise consequences of these sex differences are unclear. Though speculative, it is possible that a subset of these genes or pathways to which they belong may account for some of the sex difference-related phenotypic alterations in ALS, including in the murine models.

As several studies have reported sub-populations of microglia that may be more active in pathological conditions, we performed sub-clustering on the microglia cluster (Fig. 5c). Interestingly, sub-cluster “0”, but not the others, displayed 36 significant DEGs that differed by genotype, which was obscured when analyzing all microglia together (Fig. 5a, d, Supplementary Data 1). DEGs in microglia sub-cluster 0 included genes such as *Ctss, Ptprc* (CD45), and *Lyn*, all of which have been previously implicated in microglial responses^3,41–43^. Differentially expressed pathways according to ROAST and CAMERA included the KEGG pathway “Viral protein interaction with cytokine and cytokine receptor” (Fig. 5e, f, Supplementary Data 1).

SCENIC revealed that Early growth response 1 (EGR1) and Histone deacetylase 6 (HDAC6) regulon activities were increased in sub-cluster 0 of *SOD1*^*G93A*^ microglia relative to WT (Fig. 5g, Supplementary Data 1). The EGR1 regulon consisted of genes associated with diverse signaling pathways (Supplementary Fig. 5). EGR1 has previously been implicated in regulating early microglial responses^44^. Collectively, these data suggest that microglial dysfunction is underway at the peri-onset stage of disease at age 90 days.

### Oligodendrocytes in *SOD1*^*G93A*^ lumbar spinal cord display increased *Stat3* expression and predicted STAT3-dependent transcriptional responses

**Fig. 6:**
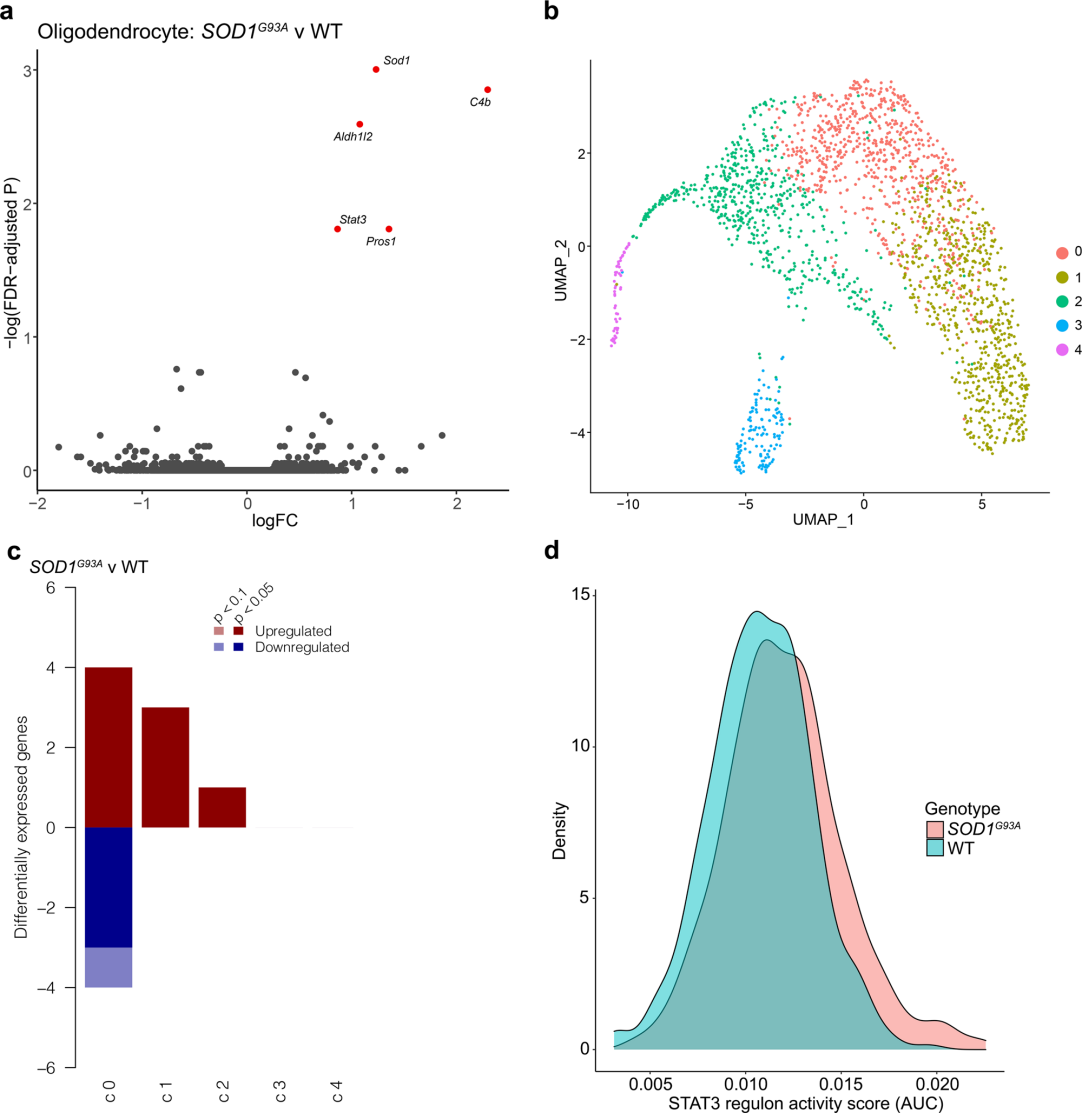
Oligodendrocytes display increased STAT3-dependent responses in *SOD1*^*G93A*^ mice at peri-symptomatic onset stage of disease. **a** Volcano plot of DEGs between genotypes in oligodendrocytes. Genes with FDR < 0.1 are colored red (up-regulated) or blue (down-regulated). **b** UMAP plot of oligodendrocyte sub-clusters. **c** Bar chart summarizing the number of DEGs across sub-clusters (FDR < 0.1). Positive and negative *y*-axis values indicate number of up-regulated and down-regulated genes, respectively. **d** Density plot of STAT3 regulon activity scores for oligodendrocyte sub-cluster 0. *N* = 3 independent mice per genotype per sex.

Differential expression analyses revealed 5 DEGs between *SOD1*^*G93A*^ and WT oligodendrocytes: *Sod1, C4b, Aldh1l2, Pros1*, and *Stat3* (Fig. 6a, Supplementary Data 1). Neither ROAST nor CAMERA detected significant alterations in Reactome or KEGG gene sets (Supplementary Data 1). As previous reports have suggested that oligodendrocytes may initiate a re-myelination response in ALS, we next considered that sub-populations of oligodendrocytes may have distinct responses^45^.

We sub-clustered oligodendrocytes and found that the largest sub-cluster of oligodendrocytes was responsible for the majority of the DEGs identified in the collective cell type (Fig. 6b, c, Supplementary Data 1). This sub-cluster of oligodendrocytes additionally displayed significant up-regulation of the KEGG pathway “Signaling pathways regulating pluripotency of stem cells” and significant alteration of the KEGG pathway “Complement and coagulation cascades” (Supplementary Data 1). SCENIC analysis of this cluster revealed significant activation of Signal Transducer and Activator of Transcription 3 (STAT3) regulon activity including target genes involved in oligodendrocyte maturation and myelination *Igf1r* and *Raf1* (Fig. 6d, Supplementary Fig. 6, Supplementary Data 1)^46,47^. Altogether, these data reaffirm previous studies indicating that while large transcriptional changes in oligodendrocytes do not occur until later stages of *SOD1* associated pathology, it is plausible that incipient STAT3-dependent transcriptional programs are emerging during peri-symptomatic onset of pathology^33^.

### Comparison with previously published spatial transcriptomics findings

Previous spatial transcriptomics analyses in this model suggested glial dysfunction as early as post-natal day 30^32^. Hence, we compared the present *SOD1*^*G93A*^ vs. WT cell type-specific alterations with genes significantly altered in various anatomical locations, as previously reported. This effort revealed that the greatest overlap between the present snRNA-seq and earlier spatial transcriptomics analyses was between astrocyte DEGs identified in the present study, followed by motor neuron DEGs, and genes significantly altered in the lumbar spinal cord ventral horn at age day 120; however, overlap was detected even at age day 30 (Supplementary Fig. 7a–c)^32^. In fact, microglia DEG *Ntng1*, motor neuron DEG *Grip1*, alongside astrocyte DEG *Sparc*, were all significantly altered in the spatial transcriptomics data by day 30. However, unifying and aggregate consequences of such alterations were not explicated in that work.

As the present studies to this point were focused on the murine model, we next sought to determine if the identification of these DEGs and predicted transcriptional regulators bore relevance to human ALS. Accordingly, we turned to a human ALS patient RNA-seq dataset publicly available through TargetALS.

### Human ALS patients exhibit differential transcriptional profiles congruent to pathological changes identified in individual *SOD1*^*G93A*^ cell types

**Fig. 7:**
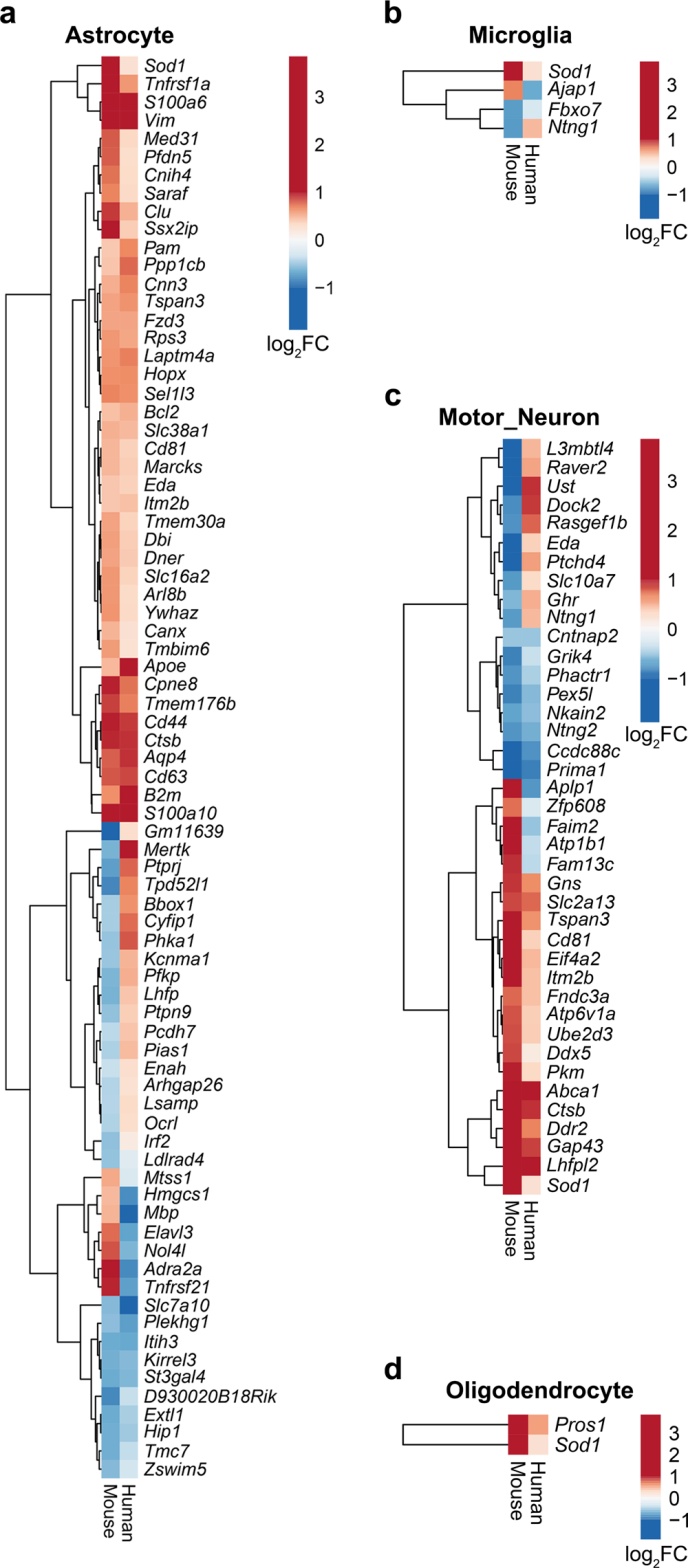
Cell type-specific *SOD1*^*G93A*^ DEGs display similarities to those identified in ALS patient cervical spinal cord relative to non-neurological control patients. Heatmaps comparing log_2_ fold change (logFC) gene expression changes in cell-specific *SOD1*^*G93A*^ vs. WT murine nuclei (Mouse) and bulk ALS patient vs. non-neurological control cervical spinal cord (Human) for murine **a** astrocytes, **b** microglia, **c** motor neurons, **d** Oligodendrocytes. *N* = 3 independent mice per genotype per sex. *N* = 76 ALS and *N* = 11 non-neurological control patients.

**Fig. 8:**
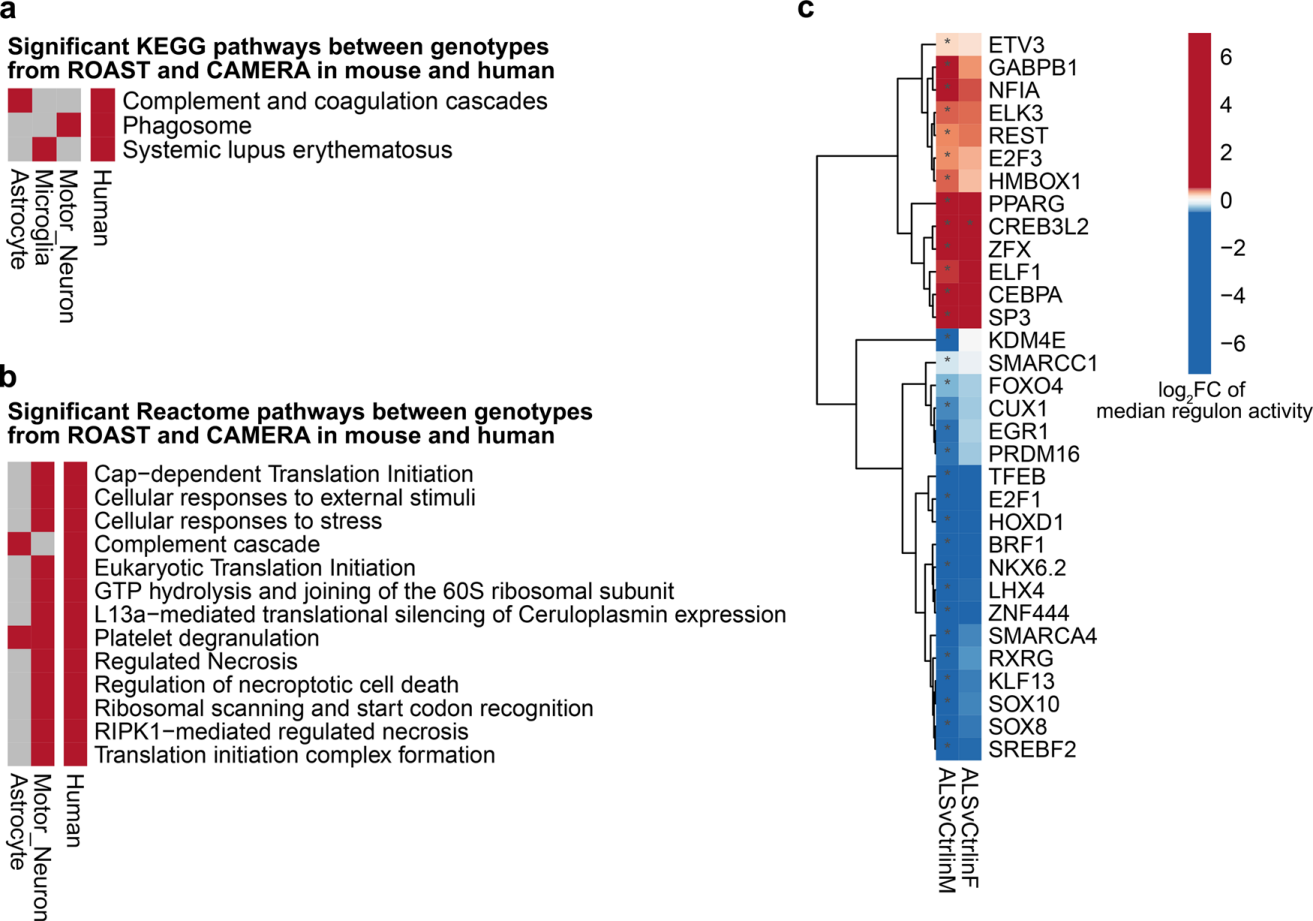
Complement-associated pathways and cell death-associated pathways are identified in both murine cell type-specific analyses and in bulk ALS patient cervical spinal cord. Heatmaps illustrating gene sets that are differentially expressed between genotypes in murine cell types and bulk human ALS patients vs. controls by both ROAST and CAMERA for (**a**) KEGG pathways and (**b**) Reactome pathways. Up-regulation and down-regulation are indicated in red and blue, respectively (FDR < 0.05). **c** Heatmap illustrating transcription factors, identified by SCENIC, whose regulon activities differ by diagnosis (FDR < 0.05). In **a**, **b** *N* = 3 independent mice per genotype per sex. In **a**–**c**, *N* = 76 ALS and *N* = 11 non-neurological control patients.

RNA-seq data from human ALS patient and non-neurological control patient cervical spinal cord tissue from TargetALS was obtained and analyzed. We began by comparing the DEGs identified between human ALS patients vs. non-neurological controls to the findings in individual murine cell types. This comparative analysis revealed three principal findings. First, direct comparison of the identified DEGs revealed broad similarity between the human bulk tissue analyses and the comparisons of *SOD1*^*G93A*^ and WT motor neurons, astrocytes, microglia and oligodendrocytes. Specifically, the number of overlapping genes for each cell type was as follows: 78/214 astrocyte DEGs, 4/7 microglia DEGs, 17/36 microglia sub-cluster 0 DEGs, 2/5 DEGs for oligodendrocytes and 40/158 DEGs for motor neurons (Fig. 7a–d, Supplementary Data 1). Comparison of the overlap of ROAST and CAMERA results in human and mouse analyses revealed several cell type-specific patterns, including up-regulation of “ Phagosome”, “Systemic lupus erythematosus”, and “Complement Cascade” between *SOD1*^*G93A*^ motor neurons, microglia and astrocytes, respectively (Fig. 8a, b, Supplementary Data 1). We next sought to examine if any predicted NicheNetR ligands were associated with available human metadata, specifically age of disease onset or disease duration, defined as the time between age at onset and age at death or tracheostomy. In human cervical spinal cord, RNA expression levels of *CDH12* were associated with age at onset, while RNA expression levels of *NRG3* and *WNT3* were associated with disease duration at FDR < 0.1 (Supplementary Data 1).

Second, SCENIC analysis of the bulk human dataset unveiled several transcription factors with altered activity in ALS that overlap with those identified in the murine cell type-specific analyses between genotypes (Fig. 8c, Supplementary Data 1). Although RCOR1 activity was not significantly altered in the human dataset, the closely-associated RE1 Silencing Transcription Factor (REST) displayed increased activity in human ALS vs. non-neurological control cervical spinal cord (Fig. 8c).

Third, lipid metabolism-modifying SWI/SNF related, Matrix associated, Actin dependent Regulator of Chromatin, subfamily a, member 4 (SMARCA4) and Peroxisome Proliferator Activated Receptor Gamma (PPARG) exhibited increased activity in *SOD1*^*G93A*^ astrocytes and in ALS patients vs. respective controls (Fig. 8c). Altogether, these results suggest that *SOD1*^*G93A*^ microglia, astrocytes and motor neurons display multiple and diverse transcriptional alterations congruent with alterations detected in bulk RNA-seq data from post-mortem human patient cervical spinal cord tissue.

## Discussion

In this study, successful application of snRNA-seq analysis of lumbar spinal cord of *SOD1*^*G93A*^ mice revealed important insights into cell type-specific transcriptional alterations at a peri-symptomatic onset stage of pathology, age 90 days. Unique findings unearthed here through *in silico* modeling of cellular cross-talk revealed numerous potential ligand–receptor pairs. Several of these ligand–receptor pairs were also implicated in human ALS patient cervical spinal cord RNA-seq data. Commonalities across the putative ligands included neuronal guidance/avoidance associated molecules, cell–cell adhesion, and extracellular matrix organization. While no ALS-causative genes were differentially expressed between genotypes, other than *SOD1*, several genes previously associated with ALS were significantly altered, including *Cdh22* and *Cav2* in astrocytes and *Epha3* in motor neurons. Deletion of *EPHA3* in humans is associated with less disease risk and was significantly down-regulated in *SOD1*^*G93A*^ motor neurons, suggesting a protective response by this gene in these cells^48^. Missense mutations in *CDH22* have been reported in a cohort of familial and sporadic ALS patients, and *Cdh22* was significantly down-regulated in *SOD1*^*G93A*^ astrocytes in the present study^49^. Finally, *Cav2* was significantly up-regulated in *SOD1*^*G93A*^ astrocytes in our dataset and mutations within a human *CAV2* enhancer element associated with decreased *CAV2* expression has been recently linked to ALS risk^50^. These three genes collectively implicate perturbations in cell-cell communication between motor neurons and other cell types, including glia.

*SOD1*^*G93A*^ motor neurons displayed reduced glutamate receptor function and alterations in synapse-associated proteins alongside active cell-death and oxidative stress pathways. RCOR1, through HDAC activity, suppresses neuronal lineage-associated genes and displayed significantly increased activity in *SOD1*^*G93A*^ motor neurons^30,51^. REST, which associates with RCOR1, exhibits increased transcriptional activity in human ALS cervical spinal cord by a similar analysis^30^. While HDAC inhibition has been investigated for nearly two decades in ALS models and as a potential therapeutic intervention in patients, lack of specificity has been a prominent caveat to this approach^52,53^. Future studies investigating the targeted inhibition of the CoREST complex, perhaps via corin, may be warranted^54^.

Growing evidence has implicated astrocyte-mediated neuronal damage in promotion of disease progression^16,34^. *SOD1*^*G93A*^ astrocytes display increased oxidative stress and metabolic responses. The present data revealed an up-regulation of *Sparc*, and *Clu*, in *SOD1*^*G93A*^ astrocytes. Astrocyte *Sparc* has been shown to increase in response to extracellular glutamate levels and regulate levels of neuronal post-synaptic glutamate receptors^38^. *Clu* has been implicated in regulating synaptic function^35^.

Similar to astrocytes adjacent to demyelinating lesions, *SOD1*^*G93A*^ astrocytes exhibit increased FOS expression and activity^55^. FOS is expected to regulate *Clu*, which has been linked to synapse function. Interestingly, PPARG transcriptional activity, as inferred by SCENIC, was increased in both the human ALS patient tissue and murine *SOD1*^*G93A*^ astrocytes. As previously reported in human ALS, 4-HNE area and intensity was greatly increased in *SOD1*^*G93A*^ mice and overlapped with astrocytes, which is notable as previous studies indicated that 4-HNE can increase PPARG activity^56,57^. In this context, regulation of PPARG may suggest processes driving attempts at re-myelination, as lipid and cholesterol metabolism are essential for these processes, which may be important in astrocyte biology^58,59^. Hence, further study is warranted to determine if selective ablation or activation of these transcription factors within these cells might ameliorate disease progression by enhancing protective functions and/or preventing deleterious transitions to a more reactive state.

In addition, *C4b* expression has been recently reported in cuprizone-induced demyelination associated oligodendrocytes and within amyloid plaque-associated oligodendrocytes^60,61^. *C4b* expression and protein release from oligodendrocytes has been posited to enhance the aggregation of amyloid beta peptide, which may conceivably alter the dynamics of protein aggregation in the ALS spinal cord^60^. Here we observed increased expression of *C4b* by oligodendrocytes at 90 days of age alongside re-myelination associated genes such as *Stat3*^62^. In fact, a sub-population of oligodendrocytes displayed significant up-regulation of a STAT3 regulon and up-regulation of a stem cell response gene set. Our investigation here illustrates that oligodendrocytes are responding early to neuronal stress and up-regulating pro-damage and pro-repair genes simultaneously prior to overt neurodegeneration. This dual-edged signature may underlie the previous reports of failed oligodendrocyte repair mechanisms in ALS^45^.

It has been proposed that microglia undergo phenotypic transitions during neurodegenerative processes in mice^41,42^, which are mediated, at least in part, through triggering receptor expressed on myeloid cells 2 (TREM2) signal transduction^41,42^. Accordingly, the present data in *SOD1*^*G93A*^ spinal cord microglia support previous reports that these cells display a unique transcriptional response, suggestive of both protective and deleterious functions^3^. A sub-population of *SOD1*^*G93A*^ microglia exhibited alterations of gene sets and genes associated with increased systemic lupus erythematosus pathway activation, which itself encompasses both complement activation and clearance mechanisms, and viral response gene sets. Altogether, these data suggest that *SOD1*^*G93A*^ microglia at 90 days of age are undergoing a transition to a pro-inflammatory phenotype. Targeting these early response pathways might revert microglia towards pro-reparative functions.

Interestingly, microglia displayed differentially expressed genes between sexes while other cell types did not. However, these diverse genes do not have any clear overlapping functions. Thus, it is not obvious how these inherent differences between male and female microglia may or may not relate to disease-associated pathologies. Perhaps more interestingly, four genes across all cell types displayed a genotype-by-sex interaction, such that the direction and/or magnitude of differences in expression between genotypes depended on the sex of the mice. Three of these genes in motor neurons are involved in cytoskeletal organization, synapse structure and extracellular matrix (ECM) composition. Polymorphisms in human *FRMPD4* have been previously linked to sex differences in schizophrenia and mutations in *FRMPD4* can cause an X-linked intellectual disability^63,64^. *Frmpd4* has been reported to regulate the formation of glutamatergic synapses and dendritic spines^65^. In the present study, *Frmpd4* expression is lower in male *SOD1*^*G93A*^ motor neurons relative to all other groups. Male patients are more likely to develop ALS at younger ages than are female patients, however, the underlying cause of these differences is not kwown^66^. Further investigation into the consequences of these sex differences and perturbation of *Frmpd4* will be necessary to determine if this contributes to the earlier incidence of pathology in male *SOD1*^*G93A*^ mice^67^. Altogether, these considerations lend credence to the requirement to include both sexes in studies investigating therapeutic opportunities in ALS.

**Fig. 9:**
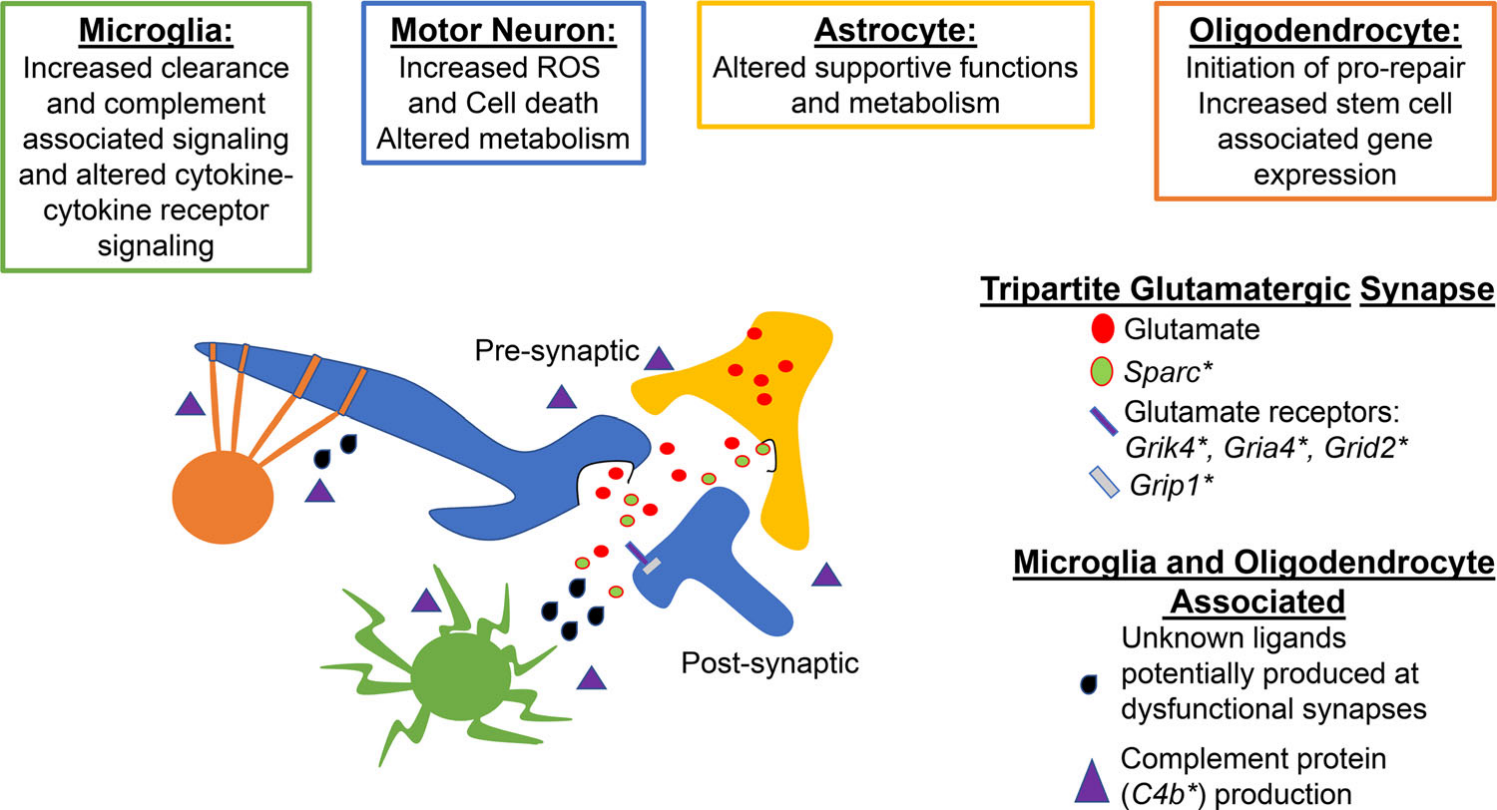
Tripartite synapse alterations coupled with failure of clearance and repair mechanisms exacerbate stress responses and perturb intercellular communications. * denotes a significant DEG identified in either astrocytes, microglia or motor neurons. Green cell = microglia. Blue = motor neuron. Yellow = astrocyte. Orange = oligodendrocyte.

In summary, we propose a model in which integrated dysregulation of motor neurons, astrocytes, microglia and oligodendrocytes is evident at a time point prior to overt ALS-associated symptoms. Specifically, this model surmises that the tripartite synapse and complement activation, exhibited by multiple cell types, such as astrocytes, microglia and oligodendrocytes, coupled with impaired reparative responses, particularly in oligodendrocytes, collectively exacerbate intrinsic stress responses and perturb extrinsic intercellular communications among motor neurons, microglia, oligodendrocytes and astrocytes (Fig. 9). The implications of these findings are clear: targeting a single mechanism to improve synaptic function, or providing discrete adaptive glial support, may not be sufficient to re-wire this complex and evolving system. Interestingly, at least a subset of these signals was detected in murine spatial transcriptomics data as early as age day 30, suggesting that once these processes begin, they continue unimpeded. Some of the identified pro-reparative responses, even at the stage of peri-onset symptomatology, are immersed in a highly reactive and pro-damage setting and, ultimately, appear to become engaged in a futile cycle in which reparative responses are overwhelmed and ineffectual. Finally, some of these findings were recapitulated in human ALS vs. non-neurological control patient RNA-seq analyses at the levels of differential expression, gene set testing and transcription factor activity predictions, thereby suggesting that these perturbations in cell-intrinsic and intercellular communications may be provoked in human disease. This work provides a resource to prompt further investigations with single-nucleus resolution while unveiling important aspects of *SOD1*^*G93A*^ murine pathology, some of which is reflected in post-mortem tissue analysis of ALS patients.

## Methods

### Animals

All experiments were performed on the following *Mus Musculus* strains at 90 and 120 days of age: B6.Cg-Tg(*SOD1G93A*)1Gur/J (JAX Stock No:004435) and C57BL/6J littermate controls^6,68^. In accordance with high-copy number transgenics, mice were checked for copy number alterations and any mice with alterations were not enrolled in the study. All mice were maintained under pathogen-free conditions. All mice were housed in a temperature (19–23 °C) and humidity (30–70% relative humidity)-regulated environment with 12-h light/dark cycle and were fed standard chow (Lab Diets, 5053) pellets and water ad libitum. All experiments were performed under protocols approved by the New York University Grossman School of Medicine Institute Animal Care Committee (IACUC) in accordance with international and NIH guidelines.

### Single-nucleus RNA sequencing

Three male and three female *SOD1*^*G93A*^ mice and three male and three female littermate control mice were employed for these studies at age day 90. Mice were anesthetized with Ketamine and Xylazine and then underwent rapid cardiac perfusion with PBS. The spinal column was rapidly removed, and the lumbar spinal cord was retrieved. Tissue was flash-frozen in liquid nitrogen and stored at −80 °C until sample processing. Single-nucleus pools from frozen lumbar spinal cord were generated with several modifications^24^. Briefly, frozen tissue was dounce homogenized with 5 strokes of the loose pestle followed by 10 strokes with the tight pestle at 4 °C in a low sucrose buffer (0.32 M sucrose, 10 mM HEPES (pH 7.5), 5 mM CaCl_2_, 0.1 mM EDTA, 3 mM MgAc, 1 mM DTT) with 0.1% Triton X-100 added. The crude nuclei prep was gently mixed with additional low sucrose buffer and was subsequently passed over a pre-wet 30 μm strainer. The filtered crude nuclei prep was centrifuged at 2613 × *g* for 10 min at 4 °C. Supernatant was decanted and the pellet was re-suspended in low sucrose buffer. An additional homogenization step using a TissueRuptor II (Qiagen Inc.) set to the lowest power was completed on each sample for 15 s. High sucrose buffer (1 M sucrose, 10 mM HEPES (pH 7.5), 3 mM MgAc, 1 mM DTT) was then layered underneath the crude prep. The crude prep was centrifuged for 25 min at 2613 × *g* at 4 °C. The supernatant was decanted and re-suspended in resuspension buffer, 1% BSA in PBS with 0.2 U/μL of Ambion RNase Inhibitor (Invitrogen, Cat:AM2684), and transferred to a low-bind conical tube. The nuclei were washed of any residual sucrose by centrifugation at 500 × *g* for 5 min at 4 °C. The pellet was re-suspended in PBS containing 20 μg/mL Fc Block CD16/CD32 (Biolegend, Cat: 101301) and incubated for 5 min at 4 °C. 0.5 μg of a nuclear hashing antibody (10 µg/ml) was added to each sample and incubated for 30 min at 4 °C. Nuclei were washed three times and finally counted using a Countess Automated Cell Counter (ThermoFisher). Three pools of nuclei were generated each containing 40,000 nuclei from samples of the four experimental groups. Nuclear hashtag antibodies used included anti-Nuclear Pore Complex Proteins Hashtag Antibodies 1–4 (BioLegend, Catalog numbers: TotalSeq-A0451, TotalSeq-A0452, TotalSeq-A0453, TotalSeq-A0454).

The 10X Chromium controller (10X Genomics) with v3 chemistry was used to capture, barcode and prepare single-nucleus libraries from each of the three pools of nuclei as per manufacturer’s instructions. The resulting libraries were sequenced on a full lane of an Illumina NovaSeq 6000 S2 flow cell for average depths of 68–104 k reads per nucleus depending on the 10X batch. Illumina base call (bcl) files for the samples were converted to FASTQ files using CellRanger 3.1, and aligned to the mm10 pre-mRNA assembly using STAR 2.5.1b including the following arguments: -outSAMmultNmax -1 -outSAMunmapped Within KeepPairs -outSAMorder PairedKeepInputOrder^69^.

### Dimensionality reduction, clustering and cell type assignment

Matrices of unique molecular identifier (UMI) gene expression and nuclear hashtag oligo (HTO) counts for each gene in each nucleus were generated with CellRanger 3.1^70^. UMIs from each nucleus were assigned to individual mice using the HTODemux function on the HTO data in Seurat 4.0.3 in R 4.0.3^71,72^, and low-quality nuclei with fewer than 200 genes or greater than 10% mitochondrial data, “doublets” with ambiguous hashtag signals, and hashtag-negative nuclei were discarded, yielding 14,662 high-quality nuclei for analyses. Mitochondrial genes were discarded. The three filtered 10X batches were integrated and normalized using the sctransform method^73^. Uniform manifold approximation and projection (UMAP) dimensionality reduction was performed on the integrated data, and 61 clusters were identified using FindNeighbors with the first 100 principal components and FindClusters with a resolution of 1.6 in Seurat^74^. Cell types were assigned to each cluster using marker genes and SingleR 1.4.1, which compares the whole-transcriptome profiles of each cluster to bulk RNA-Seq expression profiles of known, purified cell types^75,76^. Clusters “6” and “15” were not initially sharply defined by marker genes and included nuclei spread throughout many other cell type-specific clusters. Thus, we performed subclustering on these cells and repeated SingleR and marker gene analyses. One resulting subcluster was assigned to microglia, whereas the others were assigned to several other cell types. To explore the diversity of neuron subtypes in the large number of neurons, we mapped our data to snRNA-seq data from Sathyamurthy *et al*. using the Azimuth reference mapping pipeline: FindTransferAnchors with SCT normalization and PCA reference reduction followed by MapQuery in Seurat^24,72,77^. Finally, we refined all cell type assignments by relabeling stray cells assigned a cell type that was counter to the vast majority of neighboring cells in the UMAP, but not in areas where cell types formed ambiguous gradients. We tested for differences in the abundance of cell types between genotypes using a quasi-likelihood framework in edgeR^78^ with library size normalization; we modeled ~ 0 + group + batch in which the group was defined as combinations of genotype and sex and tested a contrast for the mean genotype effect across sexes.

### Murine differential gene expression

Using a pseudobulking strategy in limma 3.48.3 with TMMwsp library size normalization and voom transformation, differential gene expression among genotypes and among sexes was tested separately for each cell type^79,80^. Pseudobulking entails summing expression counts across nuclei within cell type (or cluster) within each sample to generate data with similar properties to bulk RNA-seq data. A pseudobulking strategy avoids the pseudo-replication of treating each nucleus as a biological replicate and allowed us to use well-tested standard differential expression models and normalization methods^81,82^. Groups defined by combinations of genotype and sex were modeled as a fixed effect and batch was included as a covariate (~ 0 + group + batch). Contrasts were defined to test average genotype effects between sexes, average sex effects between genotypes, and the interaction of sex and genotype. Genes were filtered using the filterByExpr function prior to normalization. ROAST and CAMERA were used to test differential expression at the level of Kyoto Encyclopedia of Genes and Genomes (KEGG) and Reactome gene sets^27,28,83–85^. False-discovery rate (FDR) less than 0.1 was considered significant for gene-level differential expression tests and less than 0.05 for gene set-level tests^86^.

### Intercellular signaling and regulatory networks

We used NicheNetR 1.0 to model intercellular communication and identify upstream ligands and receptors that might explain differential gene expression in each cell type^87^. NicheNetR links ligands and receptors to target genes combining gene expression data with databases containing known signaling and gene regulatory networks. Separate analyses were performed for motor neurons, microglia, astrocytes, and oligodendrocytes, which were set as “receiver” cells in respective analyses. For each receiver cell, differentially expressed genes (DEGs) among genotypes (FDR < 0.1) in that cell type were inputted as target genes and the other three cell types were considered possible “sender” cells. Top ligands were identified using high regulatory potential scores as given by Pearson correlation. Circos chord diagrams were generated with the circlize 0.4.8 in R^88^ to summarize ligand–receptor links inferred from NicheNetR. For plotting, ligands were assigned to particular sender cell types based on maximum mean expression.

SCENIC analysis was used to identify *cis*-regulatory transcription factors that might explain differences in gene expression across genotypes using the Python implementation, pySCENIC 0.11.2^29,89^. SCENIC first considers inter-gene expression correlations to identify modules of genes coexpressed with transcription factors (TFs) across nuclei in the data set. The gene modules are then pruned to include only genes with possible TF binding sites defined by TF motif matches. The resulting TF with target gene modules are referred to as regulons. Regulons were inferred for each cell type using the GRNboost2 method and the mm10 (RefSeq release 80) cisTarget motif database, and regulon activity scores were assigned to each nucleus with the AUCell function. Regulon activity scores use “area under the curve” (AUC) to calculate whether the input gene set is enriched within the expressed genes for each nucleus. The distribution of regulon activity (AUC) scores across nuclei can be compared to test for relative differences in the expression/activity of the regulon TF and its targets. We tested for differences in regulon activity between genotypes within each sex using Wilcoxon rank-sum tests. Given that cells were treated as replicates, we considered a very low FDR-adjusted *p*-value evidence of significance (*p* < 0.005)^86^.

### Generation of STRING protein-protein interaction networks

The list of genes within each regulon was inputted into the STRING web tool located at string-db.org with the following settings: full string network; network edges: confidence; minimum required interaction score: 0.4; max number of interactors to show: 1^st^ shell: query proteins only, 2^nd^ shell: none; disable structure previews inside network bubbles; and hide disconnected nodes in the network^90^.

### Comparison with spatial transcriptomics data

We evaluated the overlap of *SOD1*^*G93A*^ vs. WT DEGs for each cell type in our study (FDR < 0.1) with DEGs in various tissue regions at multiple time points from a recently published spatial transcriptomics study of *SOD1*^*G93A*^ lumbar spinal cord tissue (Bayes factor > 3)^32^.

### Human differential gene expression

Raw bulk RNA-seq data of cervical spinal cord and de-identified metadata were obtained from The Target ALS Multicentered Postmortem Tissue Core, the New York Genome Center for Genomics of Neurodegenerative Disease, Amyotrophic Lateral Sclerosis Association and TOW Foundation (www.targetals.org). Illumina paired-end 100-bp read data were downloaded from TargetALS (mean of 43 M reads per sample) and processed following standard quality control practices to remove low-quality reads and adapter sequence contamination (~ 4% of reads)^91–93^. Remaining high-quality read pairs were aligned to the human genome (hg19) using STAR 2.6.1d with a mean of 95% uniquely mapping. Read pair counts per gene were summed with the featureCounts function in subread 1.6.3 using the GENCODE 30 annotation release^69,94–96^. Only samples with RIN values of ≥6 were used in all analyses.

We performed differential expression analysis using limma with TMM normalization and voom transformation using groups defined by combinations of sex and disease state (model: ~0+group) and tested a contrast for the effect of ALS vs. control patients averaged across sexes. Similarly, differential expression at the level of Reactome and KEGG pathways was tested with ROAST and CAMERA. FDR < 0.05 was used as a threshold for significance. Results from human and mouse data sets were compared for one-to-one orthologs, and for gene sets with matching descriptions.

To assess the association of putative ligands predicted by NicheNetR association with human phenotypes within ALS patients, we tested for the association of gene expression with disease onset and disease duration including sex as a covariate using limma.

### Immunohistochemistry

Mice were anesthetized with Ketamine and Xylazine and then underwent rapid cardiac perfusion with PBS, followed by 4% paraformaldehyde (PFA). The spinal column was rapidly removed, and the lumbar spinal cord was retrieved. Lumbar spinal cords were washed in PBS and then drop-fixed in 4% PFA for an additional 1 h at 4 °C. After fixation, spinal cord tissue was incubated with 15% sucrose in PBS for 24 h followed by incubation with 30% sucrose in PBS for an additional 24 h. Cyroprotected spinal cords were frozen in Optimal Cutting Temperature (OCT) compound (FisherScientific, Cat: 23-730-571) and kept at −80 °C until sectioning. 8 μm thick serial sections were collected on Superfrost PLUS slides (FisherScientific, Cat: 22-037-246) using a Microm cryostat (ThermoFisher, Model: HM550). Sections were washed 3× with PBS for 5 min and then permeabilized with 0.2% Triton-X 100 in PBS for 10 min and washed 3 × with PBS. Blocking was conducted with Serum-Free blocking buffer (Dako, Cat: X090930-2) for 1 h at RT. All primary antibodies were diluted in Antibody Diluent (Dako, Cat: S3022) and applied overnight at 4 °C. Subsequently, slides were washed 3× with PBS, then incubated in secondary antibodies diluted in Antibody Diluent for 1 h at RT. As indicated, certain slides were washed and placed in a 1 μg/mL DAPI (Invitrogen, Cat: D1306) solution for 5 min at RT and washed 3× with PBS before mounting with fluorescent mounting media (Dako, Cat: S302380-2). Primary antibodies used: 0.25 μg/mL Rat anti-GFAP (2.2B10) (Invitrogen, Cat: 13-0300), 5 μg/mL Rabbit anti c-Fos (Abcam, Cat:ab190289), 1 μg/mL mouse anti- 4-hydroxynonenal (R&D Systems, Cat: MAB3249). Secondary antibodies utilized: Donkey anti-Rat Alexa Fluor 488 (Invitrogen, Cat: A-21208), Donkey anti-Mouse Alexa Fluor 546 (Invitrogen, Cat: A10036), Donkey anti-Rabbit Alexa Fluor 647 (Invitrogen, Cat: A-31573). All secondary antibodies were used at 1 μg/ml. All experiments included negative controls by omission of primary antibody.

### Imaging and quantification

Multicolor wide-field images were taken on a Leica 5500B microscope at 20× or 40× magnification, as indicated. All microscope settings were kept identical for each experiment. 2–4 images/tissue slice and 3–4 tissue slices/sample were collected for analysis. All analyses were performed with the Fiji distribution of ImageJ (NIH)^97,98^. Values from individual mice are reported in Supplemental Data 2.

### Cell area analysis

To quantify the positive area of each stain in end-stage murine and human tissues, the images underwent background removal with the rolling ball radius set to 50 within ImageJ. Images were then subjected to automated thresholding with the optimal thresholding algorithm of each signal being selected by a naïve experimenter. The following thresholding algorithms were utilized: Triangle used for GFAP and FOS, and Moments used for 4-hydroxynonenal^99–101^. Calculation of positive area was calculated per μm^2^ and the average of 6–8 images per sample was used for statistical analysis.

### Intensity analysis

Quantification of mean intensity of each stain was completed by measuring and then averaging the mean intensity of 6–8 images per animal using ImageJ. Quantification of integrated intensity within specific positive area was completed using ImageJ by restricting the intensity measurement to only areas within an automated pre-set threshold mask of another signal channel. Averages for each sample were calculated and used for statistical analysis.

### Statistics and reproducibility

Data are shown as mean ± SEM or as indicated in the figure legend. Normality of the data was assessed using the Shapiro-Wilk’s normality test. If normality assumption was met, data were subsequently evaluated by independent two-sample *t*-tests; otherwise, data were evaluated by non-parametric Mann–Whitney tests. All analyses were performed with GraphPad Prism 9 (GraphPad Software, San Diego, CA). *p* values <0.05 were used to denote statistical significance. In all cases, “N” indicates biological replicates (that is, individual mice). Technical replicates within an experiment were employed to provide a single value for a biological replicate and then statistical analyses were performed on the biological replicates.

## Supplementary information

Supplementary Information

Description of Additional Supplementary Files

Supplementary Data 1

Supplementary Data 2

## Data Availability

Murine raw (FASTQ) and aligned (BAM) sequencing data, resulting raw and normalized count data, and supporting sample- and nucleus-level metadata are publicly available through NCBI GEO accession GSE173524. All raw human RNA-seq data from Target ALS samples are publicly available via The Target ALS Multicentered Postmortem Tissue Core, the New York Genome Center for Genomics of Neurodegenerative Disease, Amyotrophic Lateral Sclerosis Association and TOW Foundation (www.targetals.org). Access can be requested by emailing ALSData@nygenome.org.

## Materials availability

All biological materials used in this study are available as indicated in the Methods section (all sources and catalogue numbers are provided).

## Code availability

All R and Python code used in data analyses and drafting of figures is available upon request.
