# Supplementary material for: Neuronal–glial communication perturbations in murine SOD1G93A spinal cord
Source: Commun Biol. 2022 Feb 28;5:177. doi: 10.1038/s42003-022-03128-y (PMC8885678; doi:10.1038/s42003-022-03128-y)
Supplement: Supplementary file 1 — Supplementary Information [file 42003_2022_3128_MOESM1_ESM.pdf]

Supplementary Information

Neuronal–Glial Communication Perturbations in Murine *SOD1*^{G93A} Spinal Cord

Michael MacLean[1], Raquel López-Díez[1], Carolina Vasquez[1], Paul F. Gugger[1],
Ann Marie Schmidt[1*]

[1]Diabetes Research Program, Department of Medicine, New York University Grossman School of Medicine, New York, NY 10016, USA

Supplementary Figures:

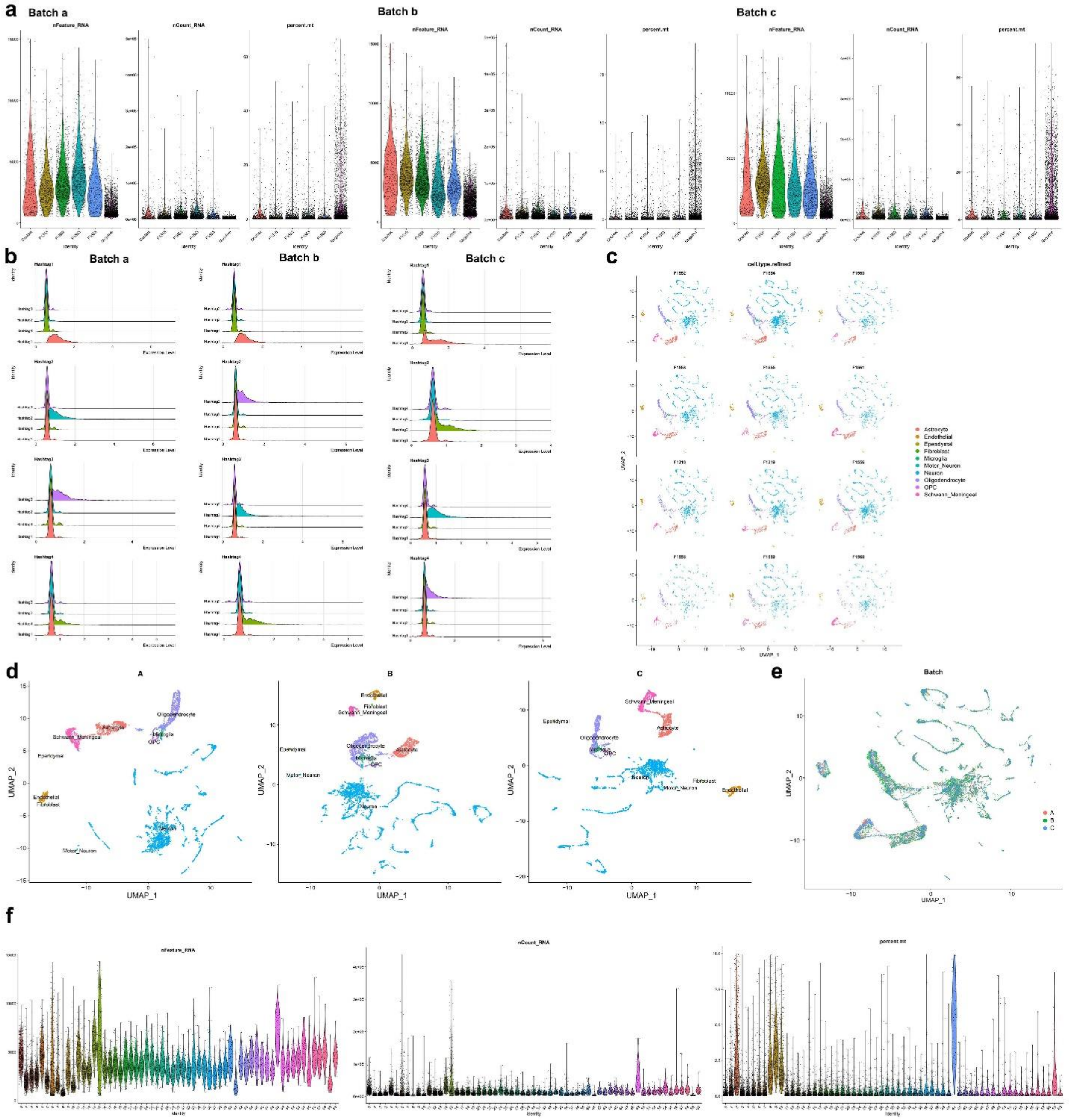

Supplementary Figure 1. Data integration of three hashtagged batches of nuclei

a. Violin plots illustrating number of total gene ("features"), total RNA counts, and percent mitochondrial data per sample or in nuclei confidently attributed to mouse samples, hashtag negative or doublet nuclei for each batch. **b.** Nuclear hashtag expression ridge plot for labeled nuclei split by unique hashtag and batch. *x*-axis: hashtag normalized hashtag expression level, *y*-axis: number of nuclei. **c.** UMAP for each sample, representing all batches (columns) and hashtags (rows). In this study each hashtag corresponds to a particular sex-genotype combination: Hashtag

1=Male WT, 2=Male *SOD1*^{G93A}, 3=Female WT and 4=Female *SOD1*^{G93A}. **d.** UMAPs for each batch after data filtering prior to data integration. **e.** UMAP of fully integrated data with nuclei colored by batch. **f.** Violin plots illustrating number of total genes (“features”), total RNA counts, and percent mitochondrial data per cluster or in nuclei confidently attributed to mouse samples, hashtag-negative or doublet nuclei. *N* = 3 independent mice per genotype per sex.

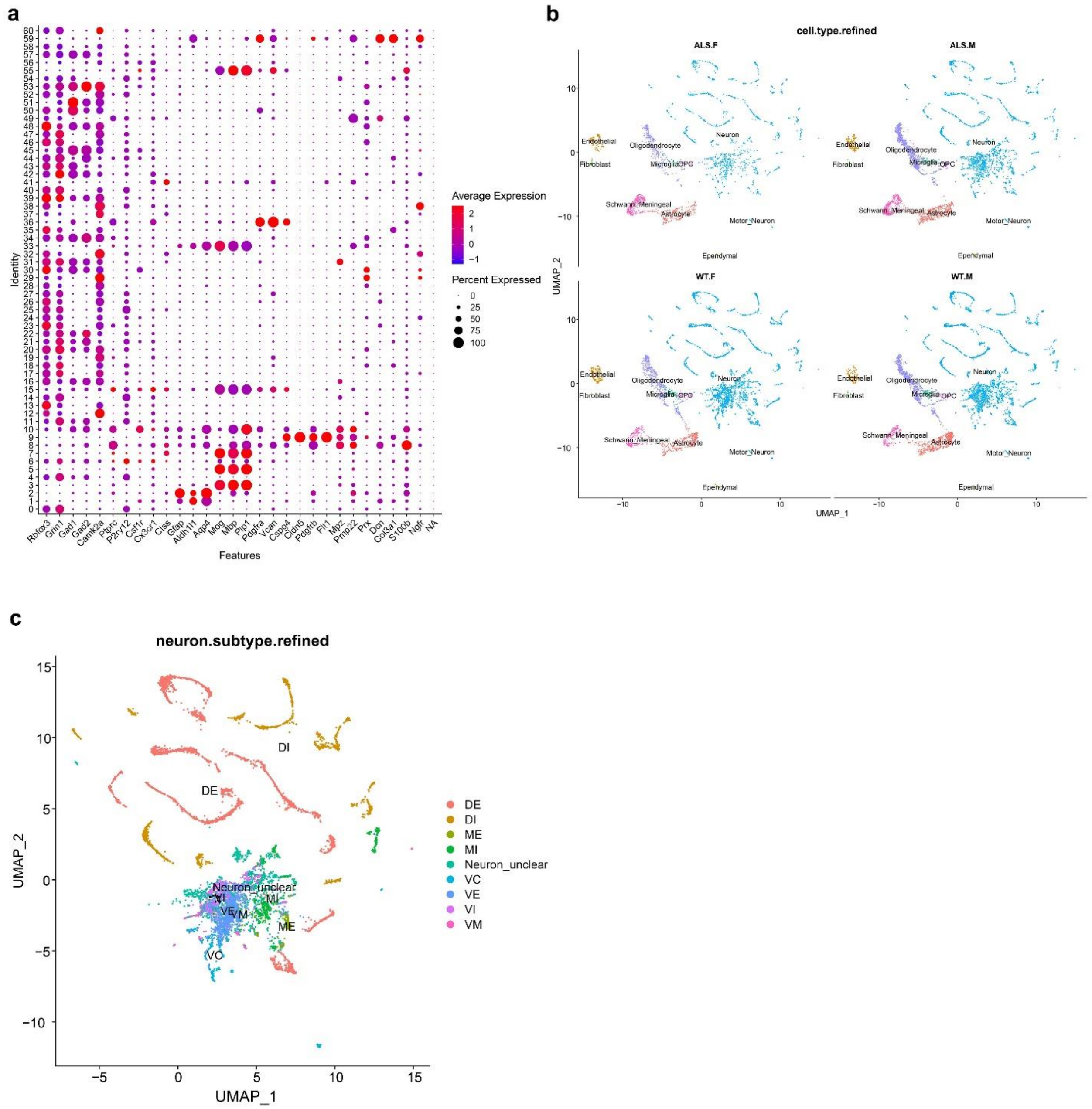

Supplementary Figure 2. Cell-type identification within integrated dataset

a. Dot plot of marker gene expression in each Seurat cluster. Clusters were labeled with cell types using a combination of marker genes and SingleR annotations. **b.** UMAP plot of cell-type labeled lumbar spinal cord nuclei split by experimental group. **c.** UMAP of neuronal sub-clusters, not including motor neurons, labeled by mapping to reference data from Sathyamurthy et al[1]. DE: Dorsal Excitatory. DI: Dorsal Inhibitory. ME: Mid Excitatory. MI: Mid Inhibitory. VE: Ventral Excitatory. VI: Ventral Inhibitory. VC: Ventral Cholinergic (non motor neurons). VM: Ventral Mixed. Unclear neurons are those not assignable to any of the above sub-types. $N = 3$ independent mice per genotype per sex.

a

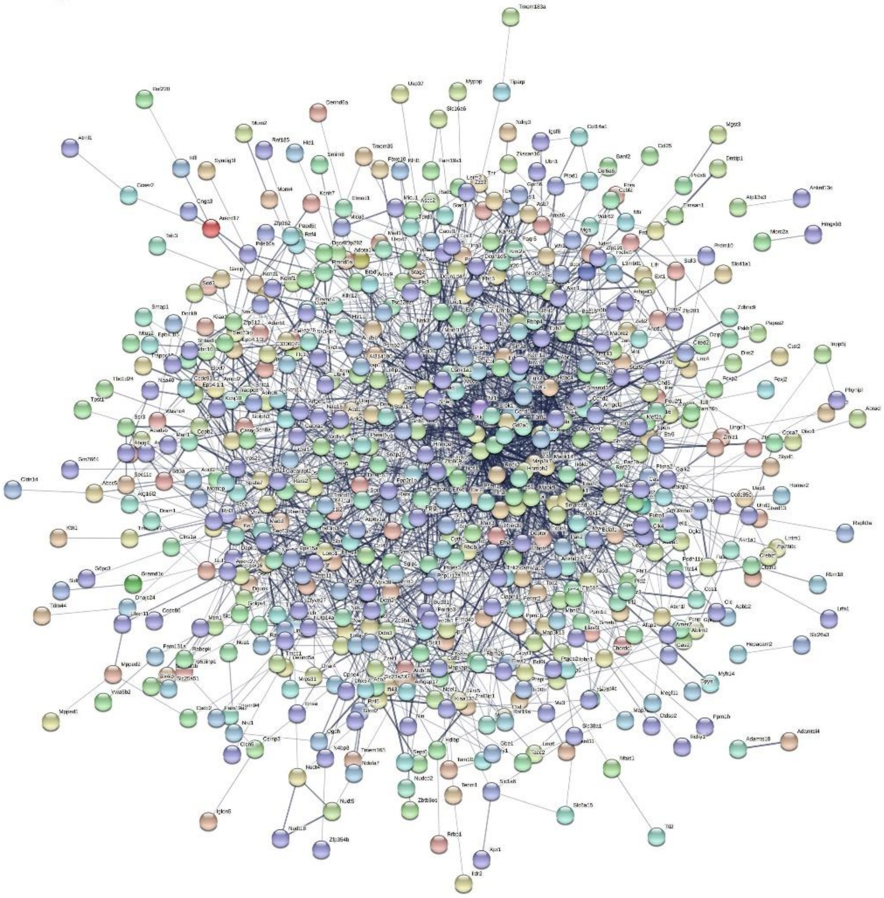

b

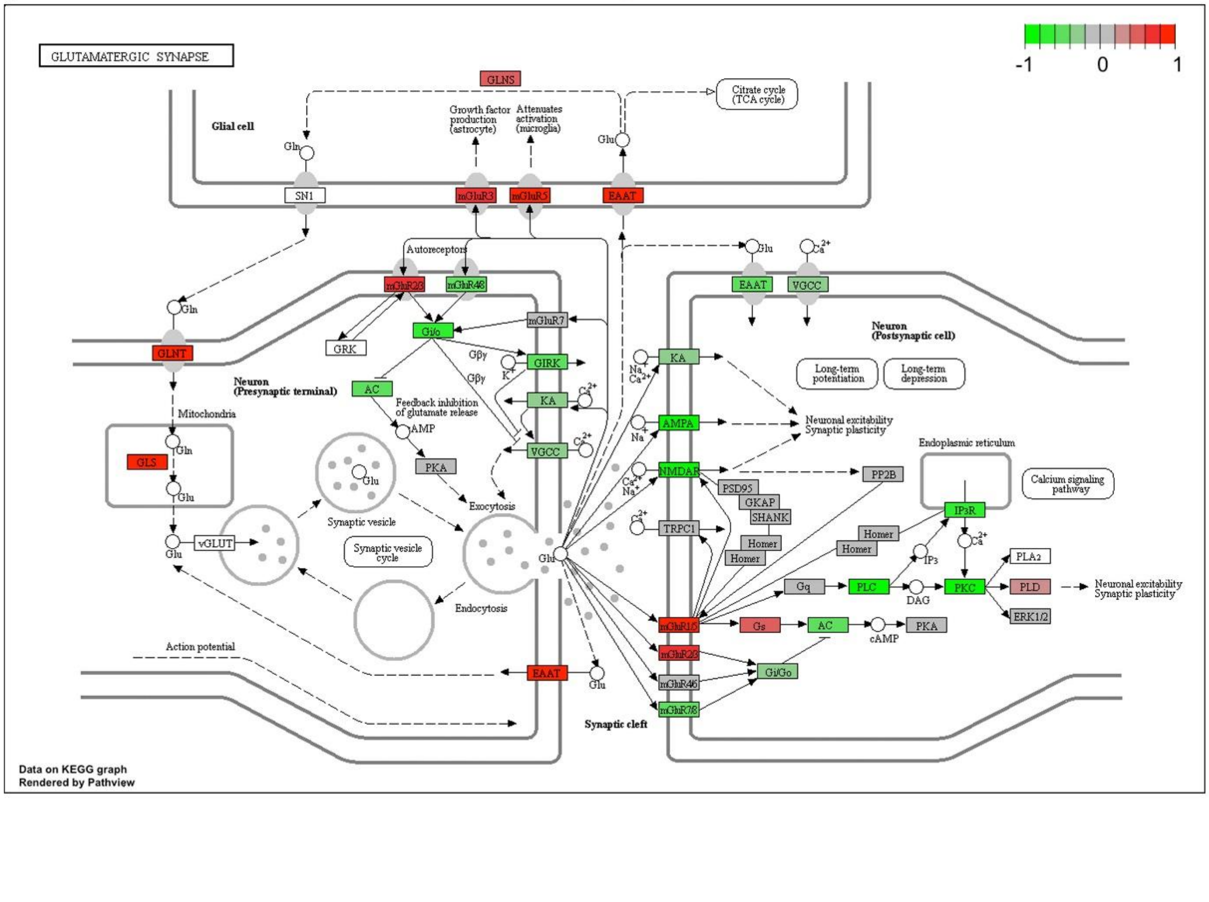
GLUTAMATERGIC SYNAPSE
-1
0
1
GLNS
Citrate cycle (TCA cycle)
Glial cell
Growth factor production (astrocyte)
Attenuates activation (microglia)
Gln
Glu
SN1
mGluR3
mGluR5
EAAT
Autoreceptors
mGluR2/3
mGluR4/8
Gln
GLNT
GRK
Gi/o
mGluR7
Neuron (Presynaptic terminal)
GIRK
KA
AC
Feedback inhibition of glutamate release
AMP
VGCC
PKA
Mitochondria
GLS
Synaptic vesicle
Exocytosis
vGLUT
Synaptic vesicle cycle
Endocytosis
Action potential
EAAT
Glu
Synaptic cleft
EAAT
VGCC
Neuron (Postsynaptic cell)
KA
Long-term potentiation
Long-term depression
AMPA
Neuronal excitability Synaptic plasticity
NMDAR
PP2B
PSD95
GKAP
SHANK
Endoplasmic reticulum
Calcium signaling pathway
TRPC1
IP3R
Homer
IP3
Gq
PLC
DAG
PKC
PLA2
PLD
Neuronal excitability Synaptic plasticity
ERK1/2
mGluR1/5
Gs
AC
cAMP
PKA
mGluR2/3
Gi/Go
mGluR4/6
mGluR7/8
Data on KEGG graph
Rendered by Pathview

Supplementary Figure 3. SOD1^{G93A} motor neurons display altered RCOR1 regulon activity alongside perturbed glutamatergic synapse associated gene expression.

a. STRING network of protein–protein interactions for genes associated with the RCOR1 regulon. Unconnected genes are not shown. **b.** Glutamatergic synapse KEGG pathway (mmu04724) colored by $\log_2$ fold change between *SOD1^{G93A}* vs. WT motor neurons. $N = 3$ independent mice per genotype per sex.

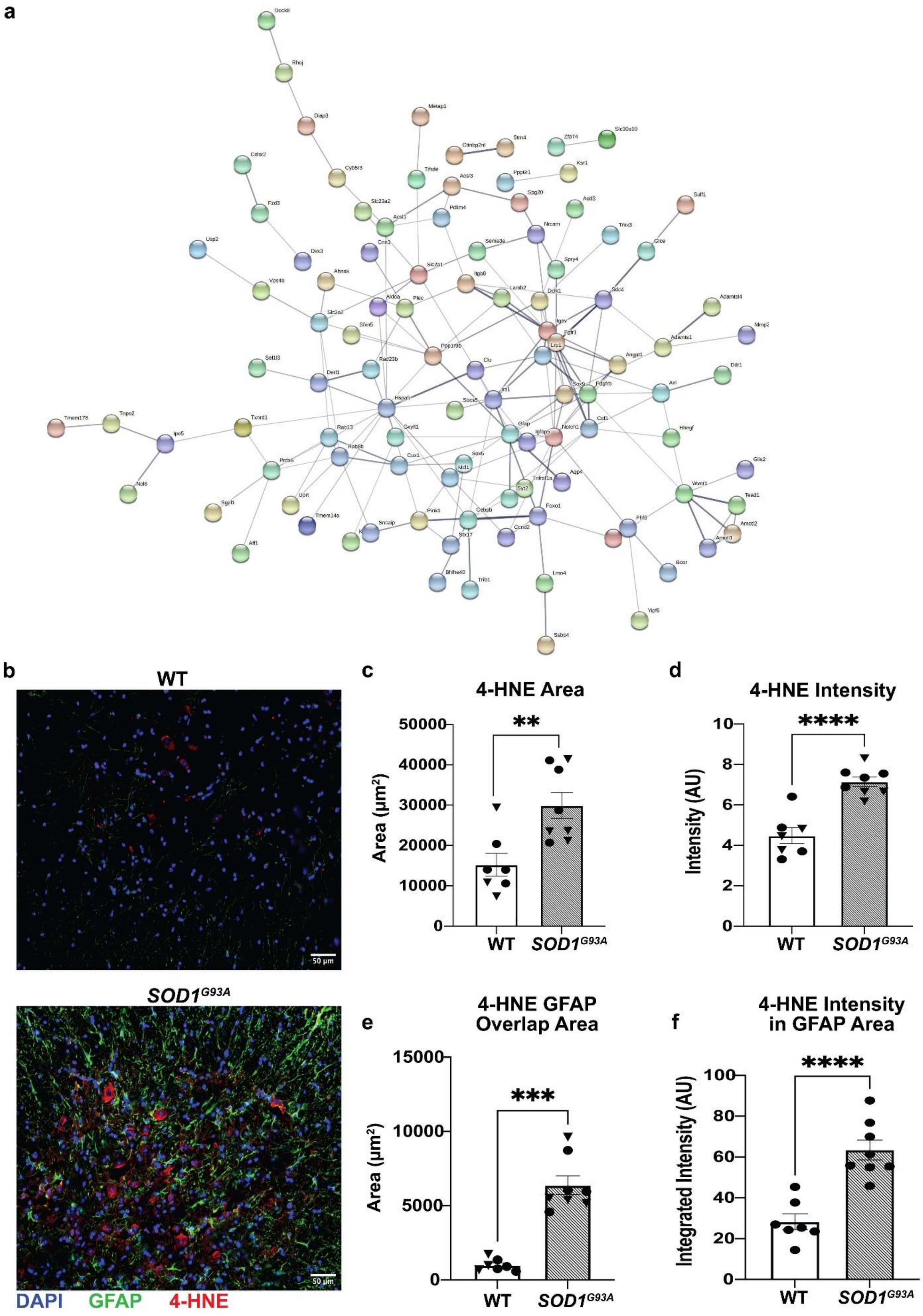
a
b
WT
50 µm
SOD1G93A
50 µm
DAPI GFAP 4-HNE
c
4-HNE Area
**
Area (µm²)
50000
40000
30000
20000
10000
0
WT
SOD1G93A
d
4-HNE Intensity
****
Intensity (AU)
10
8
6
4
2
0
WT
SOD1G93A
e
4-HNE GFAP
Overlap Area
***
Area (µm²)
15000
10000
5000
0
WT
SOD1G93A
f
4-HNE Intensity
in GFAP Area
****
Integrated Intensity (AU)
100
80
60
40
20
0
WT
SOD1G93A

Supplementary Figure 4. SOD1^{G93A} astrocytes display increased oxidative stress by age 120 days
a. STRING network of protein–protein interactions for genes associated with the FOS regulon. Unconnected genes are not shown. **b.** Representative images of DAPI, GFAP and 4-HNE in male murine *SOD1^{G93A}* and WT lumbar spinal cord at 120 days of age. **In c-f:** Quantification of **(c)** 4-HNE area; **(d)** 4-HNE intensity; **(e)** 4-HNE and GFAP overlap area; and **(f)** 4-HNE intensity within GFAP area. In **d,f**, Independent two sample two-sided *t*-test. In **c,e**: Mann-Whitney *U*-test. ** $p < 0.01$, *** $p < 0.001$, **** $p < 0.0001$. Data are presented as mean ± SEM. Specific *p* values are as follows: In c, **p=0.0059; in d, ****p<0.0001; in e, ***p=0.0003; and in f, ****p<0.0001. In **a,** $N = 3$ independent mice per sex per genotype. In **b-f**, *SOD1^{G93A}* N = 4 independent mice per sex, WT N =3 independent male and N =4 female mice. Male mice are represented by triangles; female mice are represented by circles.

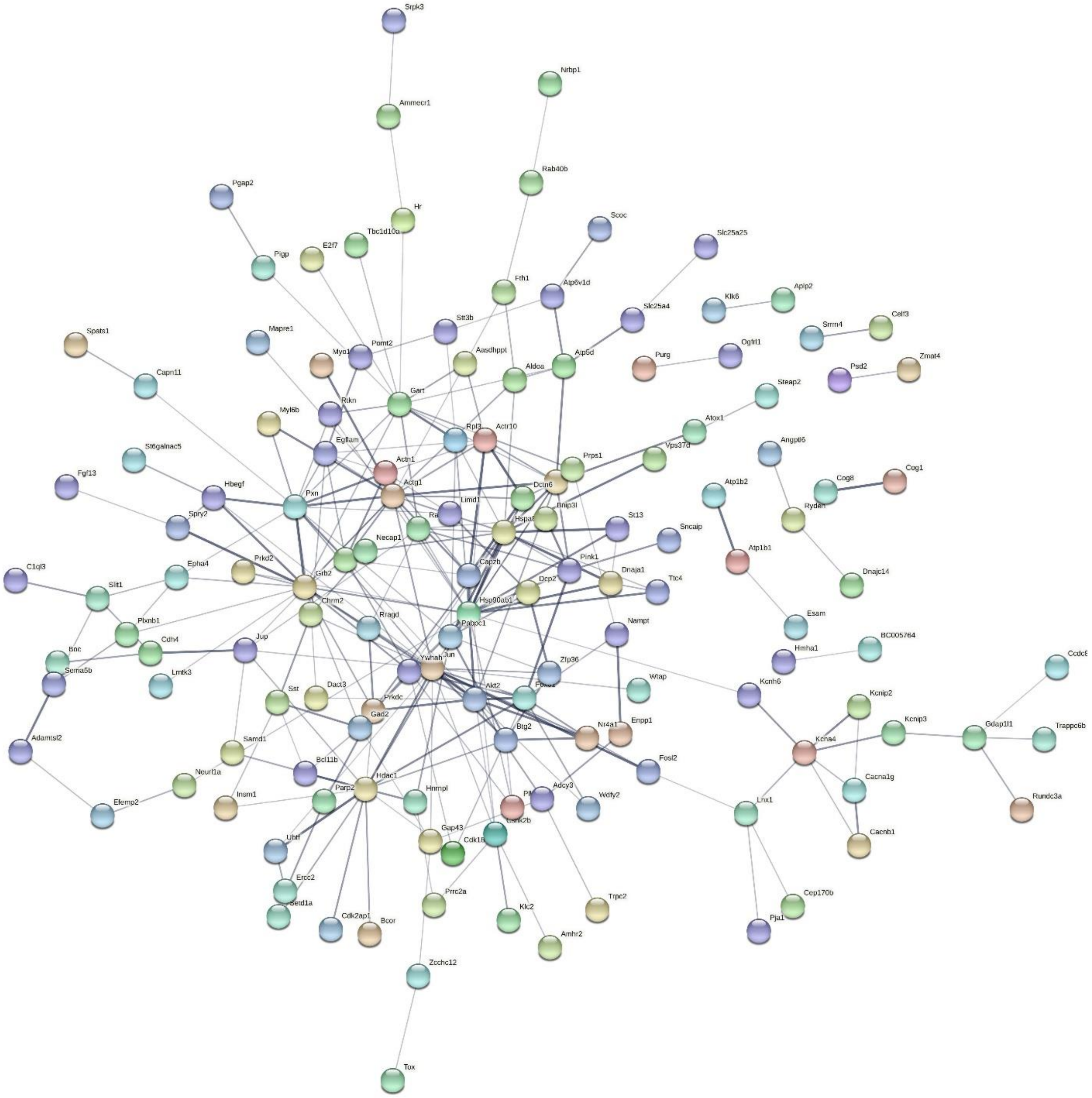

Supplementary Figure 5. Sub-cluster 0 SOD1^{G93A} microglia display up-regulated EGR1-dependent regulon activity.

STRING network of protein–protein interactions for genes associated with the EGR1 regulon. Unconnected genes are not shown. *N* = 3 independent mice per genotype per sex.

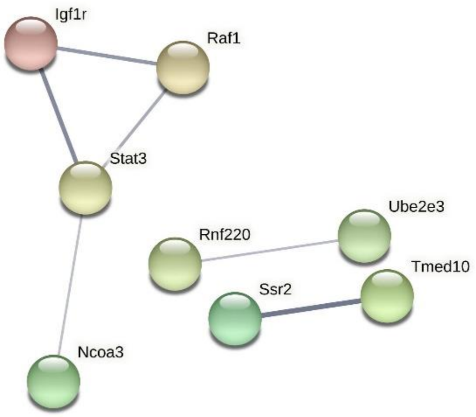

Supplementary Figure 6. Sub-cluster 0 SOD1^{G93A} oligodendrocytes display increased STAT3-dependent regulon activity.

STRING network of protein–protein interactions for genes associated with the STAT3 regulon. Unconnected genes are not shown. *N* = 3 independent mice per genotype per sex.

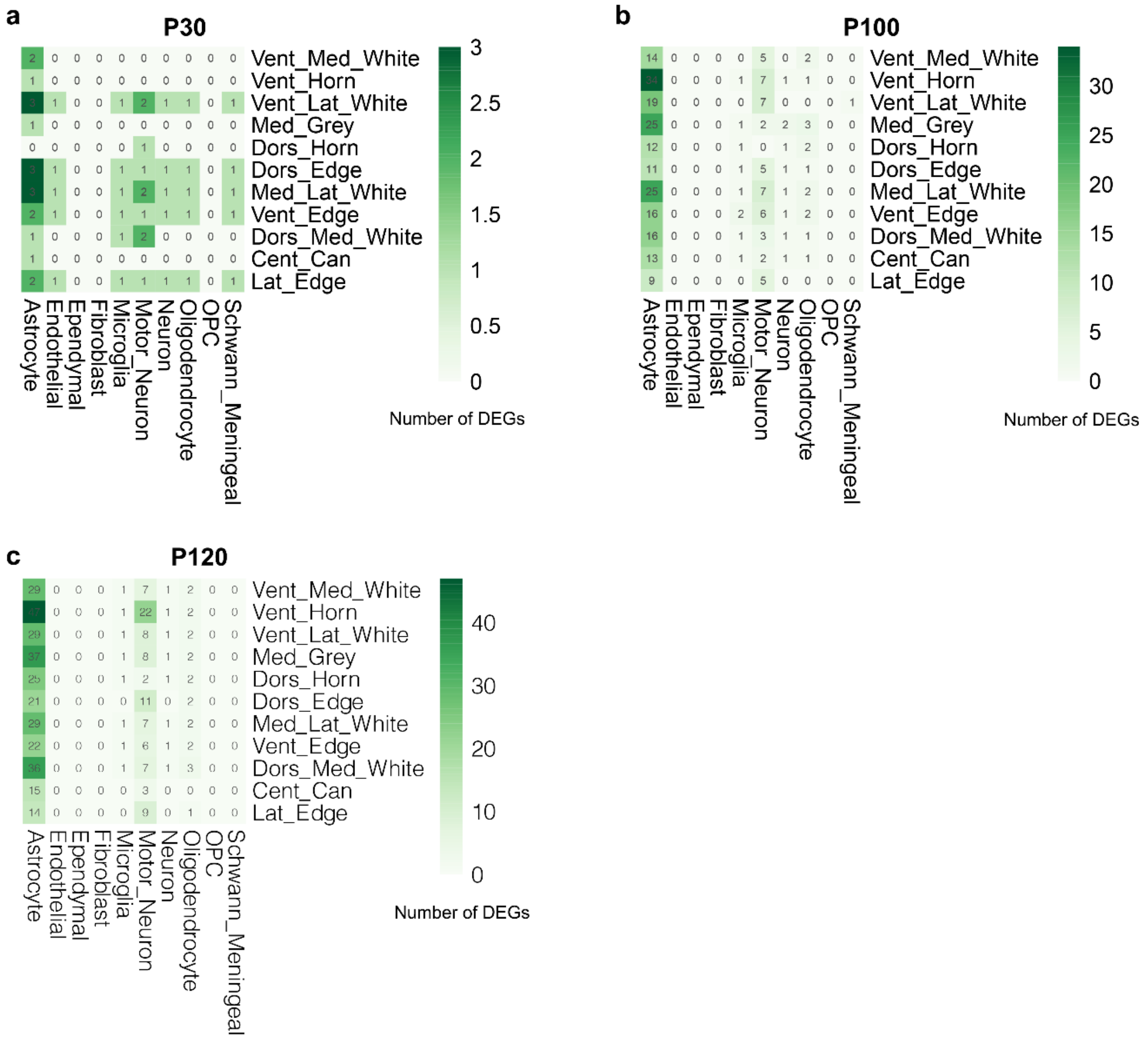

Supplementary Figure 7. Comparison of cell type-specific alterations in SOD1^{G93A} mice vs. previously reported spatial transcriptomic changes at multiple time points.

Number of DEGs overlapping between cell type-specific snRNA analyses and anatomical regions from spatial transcriptomics data from Maniatis *et al.*[2] at **a.** P30, **b.** P100, and **c.** P120. DEGs defined by FDR < 0.1 in cell type-specific analyses and Bayes factor > 3 in anatomical locations[2].

Supplementary Dataset:

Results of Limma, ROAST, CAMERA and SCENIC analyses. *N* = 3 independent mice per genotype per sex. *N* = 76 ALS patients and *N* = 11 non-neurological control patients.

Supplementary References:

1 Sathyamurthy, A. *et al.* Massively Parallel Single Nucleus Transcriptional Profiling Defines Spinal Cord Neurons and Their Activity during Behavior. *Cell Rep* **22**, 2216-2225, doi:10.1016/j.celrep.2018.02.003 (2018).

2 Maniatis, S. *et al.* Spatiotemporal dynamics of molecular pathology in amyotrophic lateral sclerosis. *Science* **364**, 89-93, doi:10.1126/science.aav9776 (2019).
