# Supplementary material for: Neuronal–glial communication perturbations in murine SOD1G93A spinal cord
Source: Commun Biol. 2022 Feb 28;5:177. doi: 10.1038/s42003-022-03128-y (PMC8885678; doi:10.1038/s42003-022-03128-y)
Supplement: Supplementary file 2 — Description of Additional Supplementary Files [file 42003_2022_3128_MOESM2_ESM.pdf]

Description of Additional Supplementary Files

File name: Supplementary Data 1

Description: *Individual mouse immunohistochemistry measures for intensity and area of GFAP, FOS, and 4-HNE reported in Figure 4 and Supplemental Figure 4.*
